# Tenascins Interfere With Remyelination in an Ex Vivo Cerebellar Explant Model of Demyelination

**DOI:** 10.3389/fcell.2022.819967

**Published:** 2022-03-15

**Authors:** Juliane Bauch, Sina Vom Ort, Annika Ulc, Andreas Faissner

**Affiliations:** Department of Cell Morphology and Molecular Neurobiology, Ruhr-University Bochum, Bochum, Germany

**Keywords:** extracellular matrix (ECM), tenascin-C, tenascin-R, myelin, myelin lesion, oligodendrocyte, regeneration

## Abstract

Oligodendrocytes form myelin membranes and thereby secure the insulation of axons and the rapid conduction of action potentials. Diseases such as multiple sclerosis highlight the importance of this glial cell population for brain function. In the adult brain, efficient remyelination following the damage to oligodendrocytes is compromised. Myelination is characterized by proliferation, migration, and proper integration of oligodendrocyte precursor cells (OPCs). These processes are among others controlled by proteins of the extracellular matrix (ECM). As a prominent representative ECM molecule, tenascin-C (Tnc) exerts an inhibitory effect on the migration and differentiation of OPCs. The structurally similar paralogue tenascin-R (Tnr) is known to promote the differentiation of oligodendrocytes. The model of lysolecithin-induced demyelination of cerebellar slice cultures represents an important tool for the analysis of the remyelination process. *Ex vivo* cerebellar explant cultures of *Tnc* ^*−/−*^ and *Tnr* ^*−/−*^ mouse lines displayed enhanced remyelination by forming thicker myelin membranes upon exposure to lysolecithin. The inhibitory effect of tenascins on remyelination could be confirmed when demyelinated wildtype control cultures were exposed to purified Tnc or Tnr protein. In that approach, the remyelination efficiency decreased in a dose-dependent manner with increasing concentrations of ECM molecules added. In order to examine potential roles in a complex *in vivo* environment, we successfully established cuprizone-based acute demyelination to analyze the remyelination behavior after cuprizone withdrawal in SV129, *Tnc* ^*−/−*^, and *Tnr* ^*−/−*^ mice. In addition, we documented by immunohistochemistry in the cuprizone model the expression of chondroitin sulfate proteoglycans that are inhibitory for the differentiation of OPCs. In conclusion, inhibitory properties of Tnc and Tnr for myelin membrane formation could be demonstrated by using an *ex vivo* approach.

## Introduction

Neurons, astrocytes, oligodendrocytes, and microglia form part of the four determining cell types of the central nervous system (CNS), whose interactions are necessary for memory formation (Hertz and Chen, 2016). Oligodendrocytes are the myelin membrane–forming cells of the CNS (Bradl and Lassmann, 2010). Due to the formation of myelin membranes, oligodendrocytes facilitate the rapid nerve conduction and insulation of axons (Hughes and Appel, 2016). In this context, conduction rates of up to 200 m/s can be reached, which are used for the complex information transfer in the CNS (Nave, 2010). A single oligodendrocyte can form up to 40 myelin membranes, which isolate many axons (Miron et al., 2011). Oligodendrocytes are postmitotic cells, and their development is characterized by proliferation of oligodendrocyte precursor cells (OPCs) (Goldman and Kuypers, 2015), migration toward target axons (Miller, 2002), and differentiation into myelin membrane–forming cells (Zhang, 2001). Furthermore, the oligodendrocyte-dependent myelination influences survival and development of neurons. Due to the importance of intact myelin, acute demyelination entails serious neurological diseases like multiple sclerosis (MS) (Franklin and Ffrench-Constant, 2017). MS is a chronic inflammatory and demyelinating disease that affects the CNS, highlighted by a demyelination of axons (Franklin and Ffrench-Constant, 2008; Hagemeier et al., 2012). MS belongs to autoimmune diseases (Sospedra, 2018), involving T-lymphocytes and activated macrophages that attack and demyelinate myelin membranes and also damage axons. Recently, it became clear that B-cells play an important role in MS by activating T-cells (Sospedra 2018). Although remyelination processes occur in the demyelinated CNS, it does not suffice for a complete regeneration (Patani et al., 2007). With regard to the clinical picture of MS, we focus on two toxicity-based demyelination models: the cuprizone and the lysolecithin demyelination model (Birgbauer et al., 2004; Kipp et al., 2009). Both models induce a reversible demyelination of axons in the absence of an immune reaction. This allows analyzing mechanisms of remyelination or factors relevant to a higher remyelination efficiency in more detail. Cuprizone is a toxic copper chelator and induces demyelination, especially in the corpus callosum and hippocampus of the CNS of rodents. Due to a reversible demyelination after withdrawal of cuprizone, a remyelination can be observed (Matsushima and Morell, 2001; Kipp et al., 2009; Ransohoff, 2012). Within the *ex vivo* model, lysolecithin induces a reversible demyelination in cerebellar slice cultures and a remyelination ensues (Miron et al., 2013). In the context of oligodendrocyte development, molecules of the extracellular matrix (ECM) have been described to alter oligodendrocyte differentiation and migration (Jakovcevski et al., 2013; Wheeler and Fuss, 2016). The ECM allows cohesion of tissue and organs and is synthesized and released by the individual cells. The matrix influences cell development, survival, proliferation, morphology, migration, and differentiation (Dityatev et al., 2010; Barros et al., 2011; Faissner and Reinhard, 2015). Earlier studies revealed that the extracellular glycoprotein Tnc has an inhibitory effect on migration and differentiation of OPCs (Kiernan et al., 1996; Czopka et al., 2009b). In demyelinated MS plaques, which are characterized by demyelination of axons, an upregulation of Tnc may mediate inhibitory influences on oligodendrocytes (Zhao et al., 2009). Along these lines, when experimental allergic encephalomyelitis (EAE) was elicited in Tnc^*−/−*^ mice, the disease course was less severe than that in the wildtype. Furthermore, proinflammatory cytokine levels and the stimulation of Th1 and Th17 cells in response to myelin oligodendrocyte glycoprotein (MOG) were less than those in the control (Momcilovic et al., 2017). In humans, tenascins belong to the glycoproteins of the ECM, with the four members of tenascin-C, tenascin-R, tenascin-X, and tenascin-W (Tucker et al., 2006). Tnc has a hexameric structure and is synthesized in early postnatal stages of CNS by neural stem cells and immature astrocytes (Bartsch et al., 1992; Garwood et al., 2004; Karus et al., 2011). The glycoprotein tenascin-R (Tnr) has a similar structure and forms dimers and trimers (Rathjen and Hodge, 2020). In contrast to Tnc, Tnr is expressed during late postnatal development of CNS in neurons and oligodendrocytes (Bartsch et al., 1992; Becker et al., 2000; Czopka et al., 2009b). Tnr promotes cell adhesion and cell differentiation of oligodendrocytes but also blocks migration of OPCs (Pesheva et al., 1997). Both tenascins exert an inhibitory influence on formation of myelin membranes. However, they act antagonistically on differentiation of oligodendrocytes. Tnc blocks differentiation, whereas Tnr is necessary for the temporal differentiation *in vitro* (Czopka et al., 2009b). Up to now, less is known about the function of Tnr. Therefore, we wanted to analyze the influence of Tnr on remyelination efficiency and on differentiation of oligodendrocytes. Here, within both demyelination models, *Tnc* ^*−/−*^ and *Tnr* ^*−/−*^ mice are used for the first time. We show that both demyelination models can be performed with *Tnc* ^*−/−*^ and *Tnr* ^*−/−*^ mice. Both glycoproteins Tnc and Tnr revealed an inhibitory influence on remyelination efficiency of oligodendrocytes in *ex vivo* explants. Furthermore, the known inhibitory effect of chondroitin sulfate on differentiation of OPCs (Karus et al., 2016; Keough et al., 2016) proved consistent with the expression pattern observed in this study.

## Materials and Methods

All experiments performed conform to the relevant regulatory standards.

### Animals and Genotyping

*Tnc* ^*−/−*^ and *Tnr* ^*−/−*^ knockout mouse (*Mus musculus*) lines were derived and maintained in the animal facility of the Faculty of Biology and Biotechnology, Ruhr University Bochum, as described (Czopka et al., 2009b). For cerebellar explant cultures, mice heterozygous for the *Tnc* and *Tnr* genes were mated, and their homozygous wildtype and *Tnc* ^*−/−*^- and *Tnr* ^*−/−*^-deficient litters were genotyped at the age of 0–3 days. In order to isolate genomic DNA, mouse tail tips were lysed in 200 µl DirectPCR® Lysis Reagent Tail (Peqlab, VWR Life Science; Cat. No. 31-101-T) containing Proteinase K (Thermo Fisher Scientific; Cat. No. EO0491) at 55°C for 30 min and centrifuged at 16,000 g for 10 s. 1 µl of lysed genomic DNA was used as a template for PCR analysis. On the one hand, the Tnc wildtype was amplified by a Tnc primer (5′-CTG​CCA​GGC​ATC​TTT​CTA​GC-3′) and Tnc Exon 1 primer (5′-TTCTGCAGGTTGGA GGCAAC-3′), resulting in a PCR product of 420 bp. The amplification of the mutant allele with the Tnc primer combined with a Tnc Neo primer (5′-CTG​CTC​TTT​ACT​GAA​GGC​TC-3′) resulted in a PCR product of 340 bp. On the other hand, the *Tnr* wildtype was amplified by a *Tnr* primer (5′-AAC​TCC​ATG​CTG​GCT​ACC​AC-3′) and an *Tnr* Exon 1 primer (5′-TTTT GGG​GAG​GTT​GAT​CTT​G-3′), resulting in a PCR product of 420 bp. The amplification of the mutant allele with the *Tnr* primer combined with a *Tnr* Neo primer (5′-ACC​GCT​TCC​TCG​TGC​TT-3′) resulted in a PCR product of approximately 429 bp. *Tnc* and *Tnr* wildtype mice will be referred to as *Tnc* ^*+/+*^ and *Tnr* ^*+/+*^, whereas *Tnc* and *Tnr* knockout mice will be referred to as *Tnc* ^*−/−*^ and *Tnr* ^*−/−*^. For cerebellar slice cultures in the presence of purified Tnr protein, homozygous NMRI-mice were used. For de- and remyelination studies in the cuprizone model, homozygous *Tnc* ^*−/−*^ and *Tnr* ^*−/−*^ mice were compared to in-house bred SV129 wildtype mice (129S2/SvPasCrl) (Charles River, Sulzfeld, Germany). All experiments and animal handling were approved by LANUV, North Rhine-Westphalia, Germany (license AZ 84-02.04-2014.A332), and conducted according to German animal protection laws.

### Lysolecithin-Induced Demyelination Model (*Ex Vivo* Model)

**FIGURE 1:**
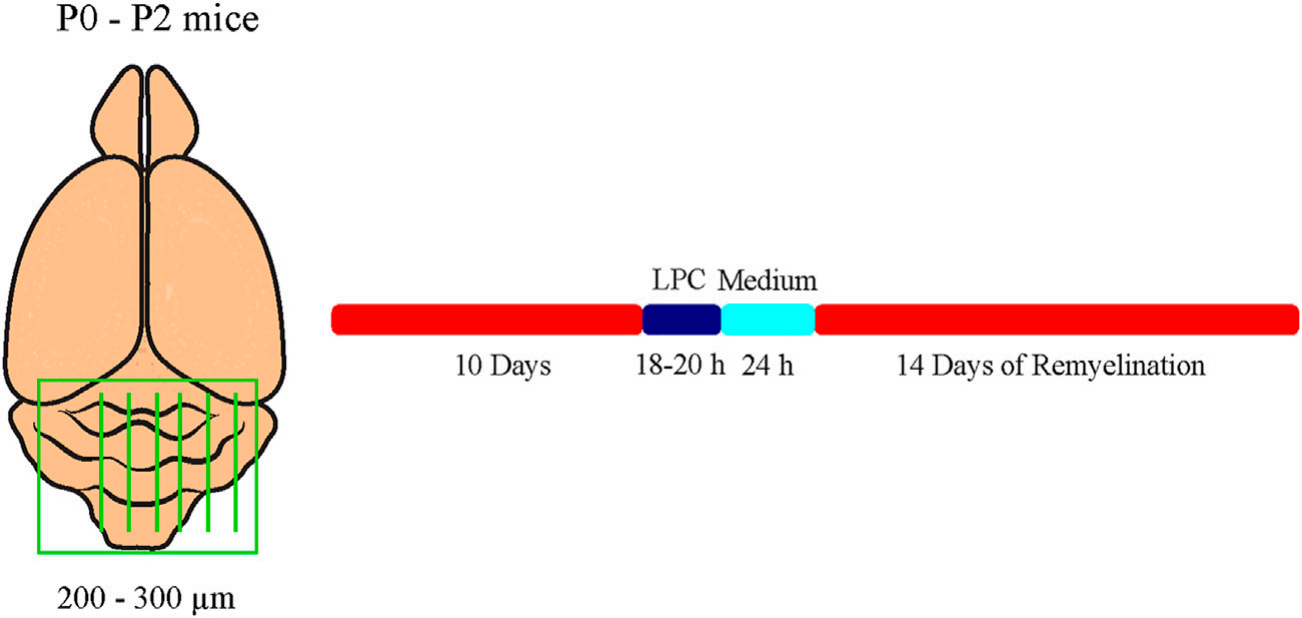
Experimental setup of the cerebellar explant cultures. Cerebella of newborn tenascin-C and tenascin-R knockout and wildtype mice pups were cut into 250–300 µm thick sagittal sections and cultivated in slice culture medium. Demyelination was induced by administration of 0.5 mg/ml lysolecithin to the medium for 16-18 h. For remyelination analysis, explants were cultivated for further 14 days. Untreated control explants (C) were kept for the whole duration of the experiment.

To analyze myelination and especially remyelination efficiency dependent on Tnc and Tnr, cerebellar slice cultures of newborn *Tnc* ^*+/+*^, *Tnc* ^*−/−*^, *Tnr* ^*+/+*^, and *Tnr* ^*−/−*^ pups were prepared as described previously (Miron et al., 2011; Zhang et al., 2011). This method represents an *ex vivo* model which allows for the application of compounds into the culture medium. In this model, the application of 0.5 mg/ml lysolecithin (Sigma-Aldrich; Cat. No. 9008-30-4) induces the demyelination of cerebellar axons (Birgbauer et al., 2004). Briefly, newborn pups were decapitated, and the brains were isolated and dissected. The cerebellum with the attached hindbrain was cut into 200–300 µm thick sagittal slices using an MCIIwain tissue chopper. Slices were placed on Millicell cell culture inserts (Merck KGaA) in slice culture medium (50% (v/v) MEM (Gibco Minimum Essential Media, Thermo Fisher Scientific; Cat. No. 15188319), 25% (v/v) Earle’s Balanced Salt Solution (EBSS, Sigma-Aldrich; Cat. No. E2888), 25% (v/v) heat-inactivated horse serum (Sigma-Aldrich; Cat. No. S9135), Pen/Strep 10 μl/ml (Sigma-Aldrich; Cat. No. P4333), and 6.5 mg/ml D-Glucose (Serva Electrophoresis GmbH; Cat. No. 22700)) in six-well culture plates. The membrane of those inserts allows gas exchange with the area and with the slice culture medium. The media were changed every 2 days. In our experimental design, two cerebellar slices a time were cultivated for each of the conditions of myelination, demyelination, remyelination, and control. After 10 days in culture, myelinated slices were fixed for staining. Demyelination was induced by administration of 0.5 mg/ml lysolecithin to the medium for 16–18 h. Thereafter, inserts of the demyelinated condition were washed and transferred to fresh medium. After an additional cultivation of 24 h, demyelinated slices were fixed. For remyelination analysis, slices were cultivated for further 14 days (Figure 1).

### Treatment of Cerebellar Slice Cultures With Exogenous Purified Tnc and Tnr

In the *ex vivo* model, remyelination efficiency was analyzed by application of purified Tnc and Tnr [diluted in phosphate-buffered saline (PBS)] in NMRI-mice to analyze its potential inhibitory influence in detail. Tnc was gained by immunoaffinity chromatography as described (Faissner and Kruse, 1990; Czopka et al., 2009b). To specify the impact on the remyelination process, Tnc was applied in a high concentration of 50 μg/ml PBS to the culture after demyelination. Regarding the concentration of Tnc in the perturbation experiments, we used 50 μg/ml to make sure that we operate in an efficient range. Concentrations down to 15 μg/ml were used in previous studies; however, these were performed with single cells, not with explants (Moritz et al., 2008). As we use Tnc purified from the postnatal mouse brain, the supplies are limited, and we could not carry out dose-response studies in this case. Therefore, we aimed at the assumed endpoint to probe the decisive issue, namely, whether Tnc interferes with remyelination. Tnr was gained by immunoaffinity purification from adult *Tnr* ^*+/+*^ mouse brains as described (Czopka et al., 2009b). To determine the impact on the remyelination process, Tnr was applied in concentrations of 10 μg/ml and 20 μg/ml PBS to the cultures after demyelination. Tnr was isolated from the adult mouse CNS and was also only available in limited amounts. Cultivation was performed as described above, but Tnr was part of the medium during the remyelination period. For internal control, purified Tnc and Tnr were also applied to untreated explants. For further external control, we used an additional culture plate with application of 1x PBS instead of Tnr. The concentrations of tenascins are difficult to compare as Tnr occurs in dimers and trimers, whereas Tnc occurs as trimers and hexamers. If we assume that Tnr trimers (Mr 480 kD) and Tnc hexamers (Mr 1000 kD) prevailed in our samples, the multimers were used in comparable nanomolar ranges at the concentrations we applied. We assume that we saturated the inhibitory effect under these conditions.

### Imaging of Cerebellar Slices

Confocal images were obtained by using a Zeiss LSM 510 Meta microscope. Images of at least three different areas of the cerebellum were taken, whereas in the z-plane, six images were recorded at intervals of 1 µm. The ImageJ Plugin JaCoP (Bolte and Cordelieres, 2006) was used to identify Manders’ overlap coefficient (MOC), which reflects the intensities of colocalized pixels in relation to the intensities of either all red pixels or all green pixels. Moreover, we compared the measured M2 values (De Santis et al., 2021) of the different genotypes *Tnc* ^*+/+*^, *Tnc* ^*−/−*^, *Tnr* ^*+/+*^, and *Tnr* ^*−/−*^ to exclude the possibility that changed myelin formation was due to the influence of the tenascins on the axons (Supplementary Figure S2). The M2 signal therein corresponded to the red fluorescence and reflected the density of axon surfaces in the explants.

### Cuprizone-Induced Demyelination Model (*In Vivo* Model)

For myelination studies *in vivo*, eight-week-old male SV129, *Tnc* ^*−/−*^, and *Tnr* ^*−/−*^ mice were fed 0.2% (w/w) cuprizone (Sigma-Aldrich; Cat. No. 370-81-0) mixed with powdered chow for six weeks to induce demyelination (Hillis et al., 2016), followed by a diet without cuprizone for one or two weeks (Albrecht et al., 2016) to allow for remyelination, as described previously (Ulc et al., 2019). Control mice received powdered chow without cuprizone. Four different experimental groups of mice were used. At the end of the experiment, mice of each group were perfused intracardially with 20 ml PBS in deep anesthesia (800 µl 0.9% (w/v) NaCl (Thermo Fisher Scientific; Cat. No. 7647-14-5), 50 µl Xylavet (xylazine) (10 mg/ml weight) (CP-Pharma, Handelsgesellschaft mbH; Cat. No. 1205), and 150 µl ketamine (150 mg/ml weight) (CP-Pharma, Handelsgesellschaft mbH; Cat. No. 1202)), and brains were removed and cut sagittally in two halves. Then, one hemisphere was fixed with 4% (w/v) paraformaldehyde (PFA) (Carl Roth; Cat. No. 4235.1) in PBS at 4°C for 48 h, before embedding in tissue-freezing medium for cryosectioning and following immunohistochemical staining. The second hemisphere was frozen in liquid nitrogen for RNA analysis.

### Immunohistochemistry

#### Immunological Reagents

The following primary antibodies were used: APC (clone CC1, mouse IgG2b, IF: 1:100, Abcam (Ab16794) RRID:AB_443473; Cambridge, UK), MBP (mouse IgG, IF: 1:50, Bio-Rad (MCA409) RRID:AB_325004, Millipore, Eschborn, Deutschland), NF200 (rabbit polyclonal, IgG, IF: 1:200, Sigma-Aldrich (N4142) RRID:AB_477272, Chemie GmbH, Munich Germany), Olig2 (rabbit polyclonal IgG, IF: 1:500, Merck (AB9610) RRID:AB_570666, Millipore), and 473HD (rat, IgM monoclonal, IF: 1:100, Hybridoma-technique) (Faissner et al., 1994a). Species-specific secondary antibodies coupled to Cy2 AF488 (1:250) and Cy3 (1:500) were obtained from Dianova GmbH (Hamburg, Germany).

#### Immunohistochemical Staining

After 2 days, PFA-fixed brain halves were dehydrated in 30% (w/v) sucrose (J.T. Baker; Cat. No. 4072-01) before embedding in tissue-freezing medium (Leica Biosystems; Cat. No. 14020108926) on dry ice. For immunohistochemical staining, cryosections with a slice thickness of 14 µm were used. Here, we focused sagittal sections on the area of the corpus callosum (CC). To permeabilize the tissue, brain sections were first boiled with blocking solution in 0.01 M citrate buffer before incubation (PBS+ 1% (w/v) BSA (Carl Roth GmbH & Co. KG; Cat. No. 8076.2) + 0.1% (v/v) Triton-X 100 (AppliChem; Cat. No. A4975,0500) + 5% (v/v) goat serum (Abcam; Cat. No. ab7481)) for 1 h at RT in a humid chamber. Individual primary antibodies (MBP, 473HD, Olig2, and CC1) were diluted in blocking solution and incubated at 4°C overnight. After three consecutive washing steps in PBS, secondary antibodies were diluted in PBS/A (PBS + 0.1% (w/v) BSA) and incubated for 2 h at RT. Finally, stained cryosections were washed three times with PBS and mounted using ImmuMount. For luxol fast blue–periodic acid Schiff’s reagent staining (LFB–PAS-staining), LFB (Thermo Fisher Scientific; Cat. No. 1328-51-4) was used to stain myelin blue and PAS to stain axons red. At the beginning, *in vivo* sections were dehydrated in a graded alcohol series (30% (v/v), 70% (v/v), 90% (v/v), 96% (v/v) ethanol) and stained in 1% LFB solution in 96% (v/v) ethanol (Carl Roth; Cat. No. 64-17-5) for 24 h at 60°C. Afterward, cryosections were rinsed in 96% (v/v) ethanol followed by incubation in 0.05% (w/v) lithium carbonate (LiCO_3_) (Sigma-Aldrich; Cat. No. 554-13-2) for 30 s. Next, the sections were washed in 70% (v/v) ethanol for 30 s, which reduces the background staining, and in A. dest. for 5 min to block differentiation. Then, staining was followed with 1% (w/v) periodic acid (7 min) and afterward with Schiff’s reagent (Carl Roth; Cat. No. 1789) for 20 min. Subsequently, the sections were washed three times with A. dest. before they were stained in hematoxylin for 3 min for making the cell nuclei in tissue visible. After further dehydration in a graded alcohol series, cryosections were mounted using Euparal mounting medium (Carl Roth; Cat. No. 1993). For the immunohistochemical staining of cerebellar explant cultures, slices were fixed in 4% (w/v) PFA in PBS for 1 h at RT and afterward rinsed three times in PBS for 10 min. For staining, slices were permeabilized and blocked in 3% (v/v) heat-inactivated horse serum (HS), 2% BSA (w/v), and 0.5% (v/v) Triton-X 100 (blocking buffer) for 1 h at RT. Primary antibodies were diluted in blocking buffer and incubated at 4°C overnight. Secondary antibodies were diluted for 3 h at RT, followed by two washes in PBS for 10 min and one wash in PBS for 1 h. Slices were mounted on glass slides using ImmuMount. Confocal images were collected by using a Zeiss LSM 510 Meta microscope at intervals of 1 µm. The ImageJ Plugin JaCoP (Bolte and Cordelieres, 2006) was used to identify the individual Manders’ coefficient, which describes the intensity of colocalized pixels in relation to the intensity of all red pixels. Here, the myelination index describes the percentage of myelinated axons in relation to all axons.

### Molecular Biology

#### RNA Isolation, cDNA Synthesis, and qRT-PCR

In order to monitor myelin-specific genes in the corpus callosum, the anatomical region was prepared from partially frozen brain halves of cuprizone-treated mice. Prior to RNA extraction, the isolated corpus callosum tissue was stored at −80°C. Total RNA from the corpus callosum was isolated using the GeneElute™ Mammalian Total RNA MiniPrep Kit (Sigma-Aldrich by Merck KGaA; Cat. No. RTN350) according to the manufacturer’s instructions. 0.5 µg RNA was transcribed into cDNA in a volume of 40 µl using the First Strand cDNA Synthesis Kit (Thermo Fisher Scientific; Cat. No. K1622). 6.25 ng cDNA in total was used for each qRT-PCR. All qRT-PCRs were performed on LightCycler96 (Hoffmann-La Roche AG) using the FastStart Essential DNA Green Master Kit (Hoffmann-La Roche AG; Cat. No. 064027120 01). For each condition (control, demyelination, one-week remyelination, two-weeks remyelination), cDNA samples from three different animals were prepared in triplicate. For each RT-PCR, only one myelin gene was analyzed (β-actin and ribosomal protein lateral stalk subunit P0 (RPLP0) as controls, myelin basic protein (MBP) and proteolipid protein 1 (PLP1) as myelin genes). The following primers were used: β-actin forward: 5′-cta​agg​cca​acc​gtg​aaa​ag-3′, β-actin reverse: 5′-acc​aga​ggc​ata​cag​gga​ca-3′, RPLP0 forward: 5′-cga​cct​gga​agt​cca​act​ac-3′, RPLP0 reverse: 5′-atc​tgc​tgc​atc​tgc​tg-3′, PLP1 forward: 5′-caa​gac​ctc​tgc​cag​tat​ag-3′, PLP1 reverse: 5′-agc​tca​gaa​ctt​ggt​gcc​tc-3′, MBP forward: 5′-agc​cga​ggt​ccc​att​gtt-3′, and MBP reverse: 5′-cct​cag​agg​aca​gag​tga​tgt​gtt​t-3′. In each sample, three technical replicates were measured and normalized to the expression of stably expressed genes (β-actin and RPLP0). qPCR data were analyzed with the program LightCycler^®^96 and given as values. Evaluating the primer efficiency and relative gene expression was performed with Rest 2009.

### Statistics

All results are provided as mean ± SEM if not declared otherwise. The number of performed experiments and the type of statistical tests are indicated in figure legends. The two-way ANOVA and the subsequent Tukey’s multiple-comparisons test were performed for the comparison of more than two samples. All statistical tests were run using the GraphPad Prism 7 program (GraphPad Software, San Diego, United States) and were considered as significantly different when *p* ≤ 0.05; *p*-values are indicated with * for *p* ≤ 0.05, ** for *p* ≤ 0.01, and *** for *p* ≤ 0.001.

## Results

### Faster Remyelination in Cerebella Slice Cultures of *Tnc* ^*−/−*^ Mice

**FIGURE 2:**
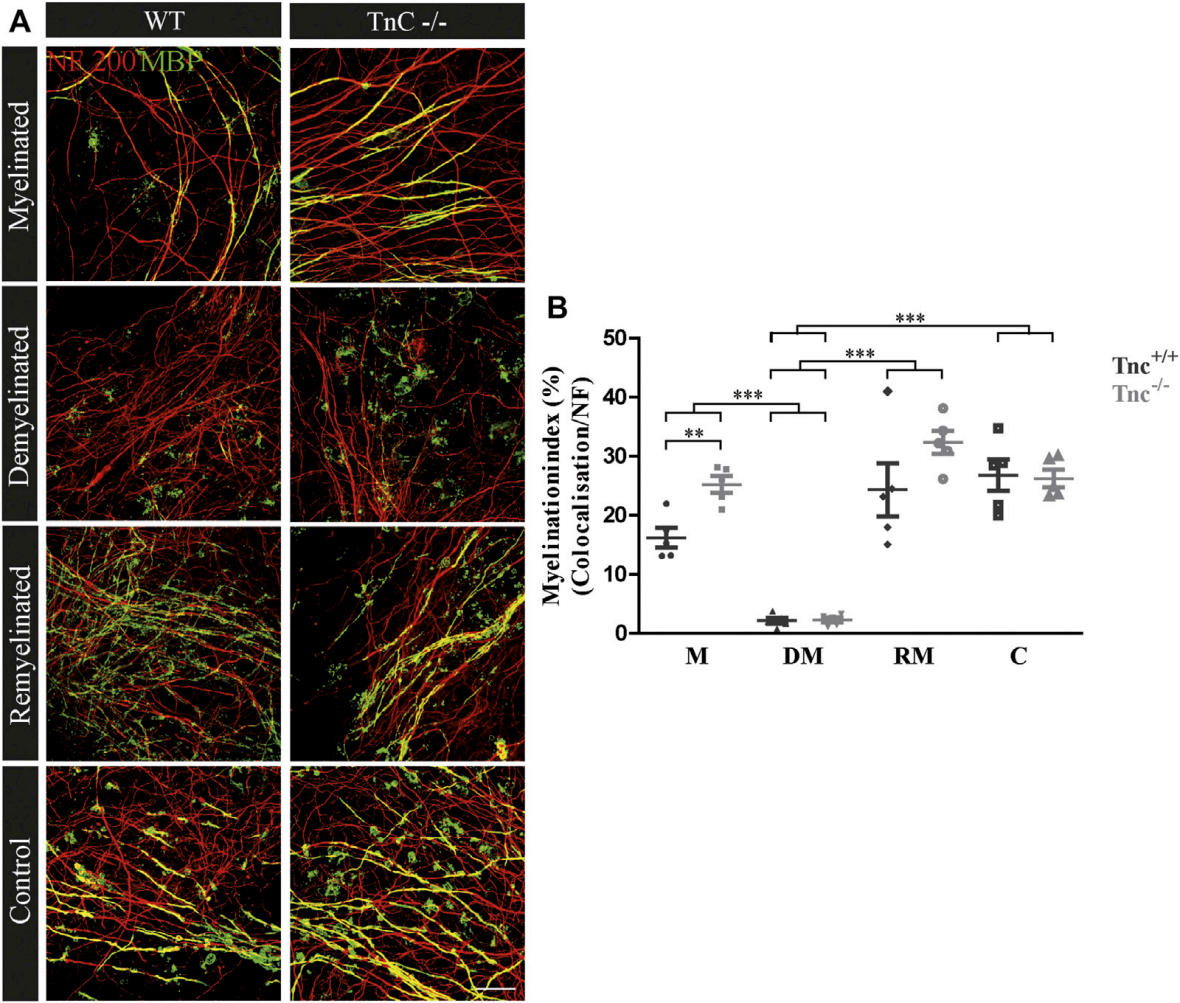
**(A,B)** Comparison of myelination and remyelination in live cerebellar explant cultures of *Tnc* ^*+/+*^ and *Tnc* ^*−/−*^ mice. **(A)** *Tnc* ^*+/+*^ and *Tnc* ^*−/−*^ cerebellar explant cultures of the conditions myelinated (M), demyelinated (DM), remyelinated (RM), and control (C) were labeled with antibodies against NF200 (red) and MBP (green) to visualize the wrapping of myelin membranes around nerve fibers. Colocalization of neurofilament and MBP appeared as yellow staining. Demyelination nearly eliminated MBP-labeling. The myelinated condition shows a highly distinctive MBP staining. An initiating remyelination is visible for both genotypes. **(B)** Quantification of myelination indices of each condition in the comparison of *Tnc* ^*+/+*^ and *Tnc* ^*−/−*^ explant cultures (M, myelinated; DM, demyelinated; RM, remyelinated; C, control). For the demyelinated condition, the quantification shows a significant reduction of the myelination index compared to the other conditions. Overall, both myelination and remyelination appeared more extensive in the absence of Tnc. All data are provided as mean ± SEM. Statistical significance was assessed using the two-way ANOVA (*p ≤ 0.05, **p ≤ 0.01, ***p ≤ 0.001) and Tukey’s multiple-comparisons test. Scale bars: 20 µm. Six independent experiments (N = 6) were performed, and three explants (*n* = 3) were analyzed per experiment for each condition.

**FIGURE 3:**
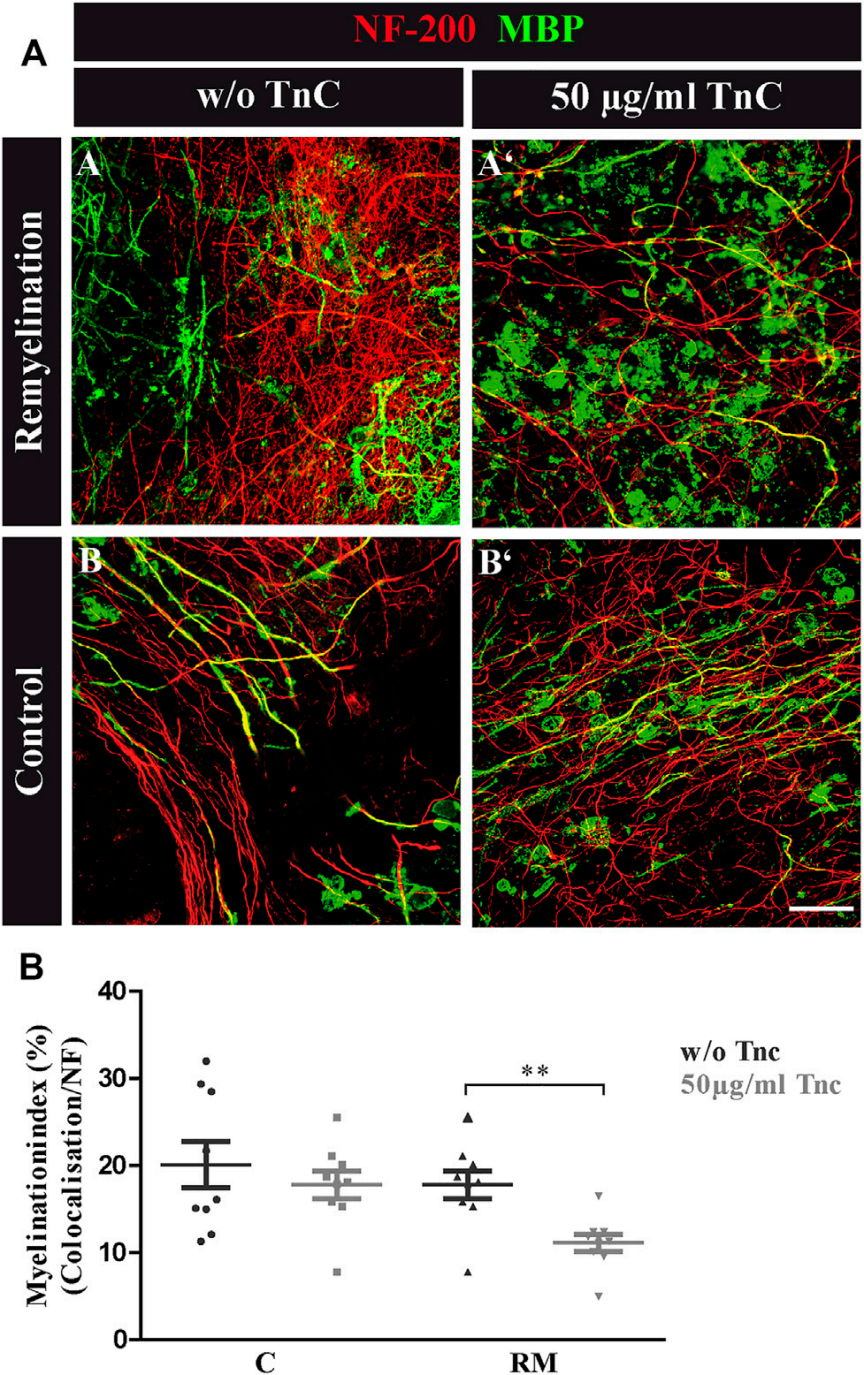
**(A,B)** Tnc inhibits remyelination in cerebellar slice cultures**. (A,A′,B,B′)** Exemplary cerebellar explants cultivated in the absence **(A,B)** or presence **(A′,B′)** of Tnc. Slices were stained for NF-200 and MBP. Tnc-treated remyelinated cultures displayed myelin membranes that did not wrap the axons throughout the whole cerebellar slice. Scale bar: 20 μm. **(B)** Evaluation of myelination index, defined as the proportion of colocalized area among the areas of complete neurofilament staining. These results indicate an impaired remyelination in Tnc-treated cultures. Data are presented as mean ± SEM, and statistical significance was assessed using an unpaired two-tailed Student’s t-test for each group (remyelinated and control). Six independent (N = 6) experiments were performed, and three explants (*n* = 3) were analyzed per experiment for each condition.

**FIGURE 4:**
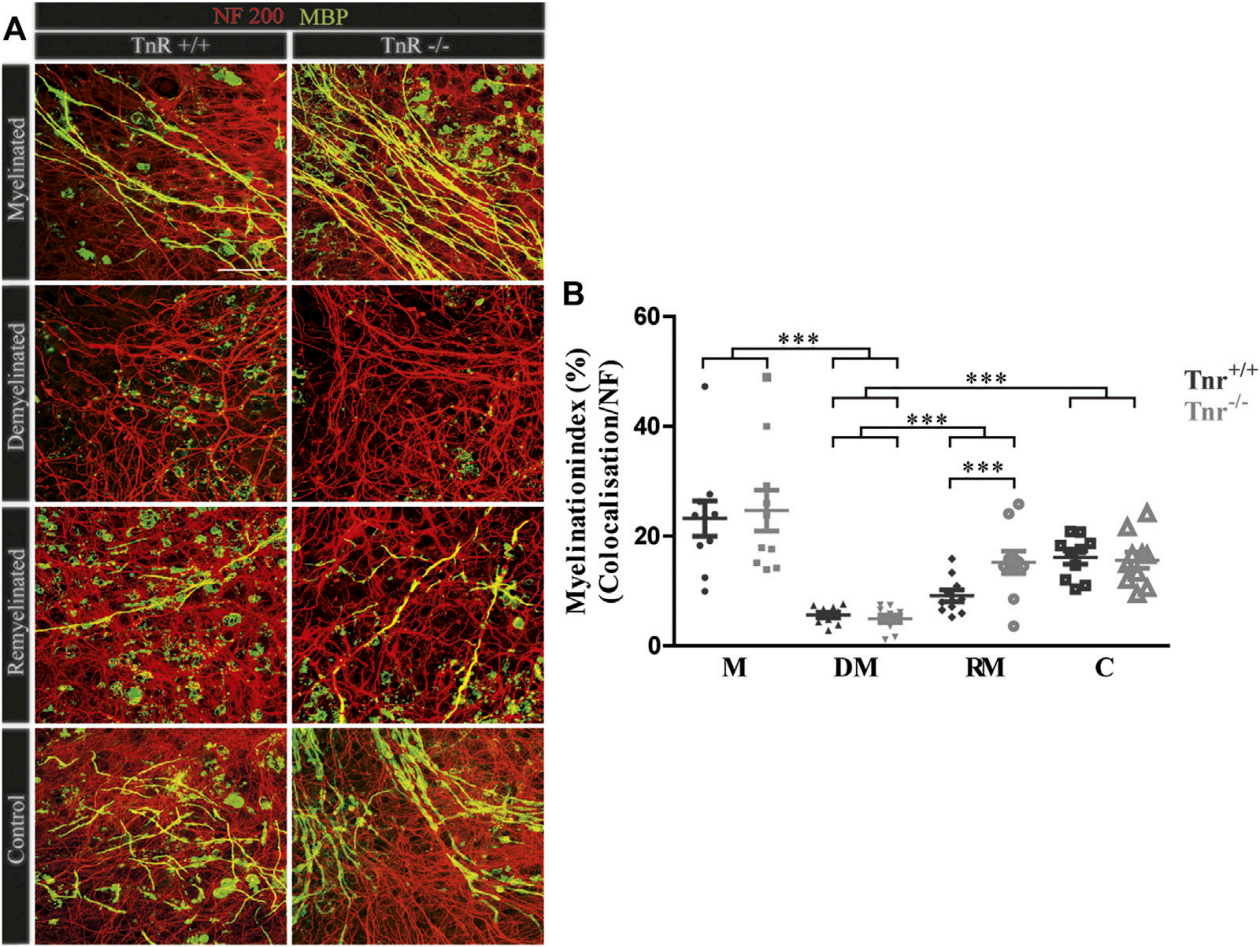
**(A,B)** Comparison of myelination and remyelination in live cerebellar explant cultures of *Tnr* ^*+/+*^ and *Tnr* ^*−/−*^ mice. **(A)** *Tnr* ^*+/+*^ and *Tnr* ^*−/−*^ cerebellar explant cultures of the conditions myelinated (M), demyelinated (DM), remyelinated (RM), and control (C) were immunohistochemically labeled with antibodies against NF200 (red) and MBP (green) to visualize the wrapping of myelin membranes around nerve fibers. Colocalization of neurofilament and MBP appeared as yellow staining. Demyelination nearly eliminated MBP-labeling. The myelinated condition shows a highly distinctive MBP staining. An initiating remyelination is visible for both genotypes. **(B)** Quantification of myelination indices of each condition in the comparison of *Tnr* ^*+/+*^ and *Tnr* ^*−/−*^ explant cultures (M, myelinated; DM, demyelinated; RM, remyelinated; C, control). For the demyelinated condition, the quantification shows a significant reduction of the myelination index compared to the other conditions. Overall, both myelination and remyelination appeared more extensive in the absence of Tnr. All data are provided as mean ± SEM. Statistical significance was assessed using the two-way ANOVA (*p ≤ 0.05, **p ≤ 0.01, ***p ≤ 0.001) and Tukey’s multiple-comparisons test. Scale bars: 50 µm. Nine independent experiments were performed (N = 9), and three explants (*n* = 3) were analyzed per experiment for each condition and each slice culture group.

**FIGURE 5:**
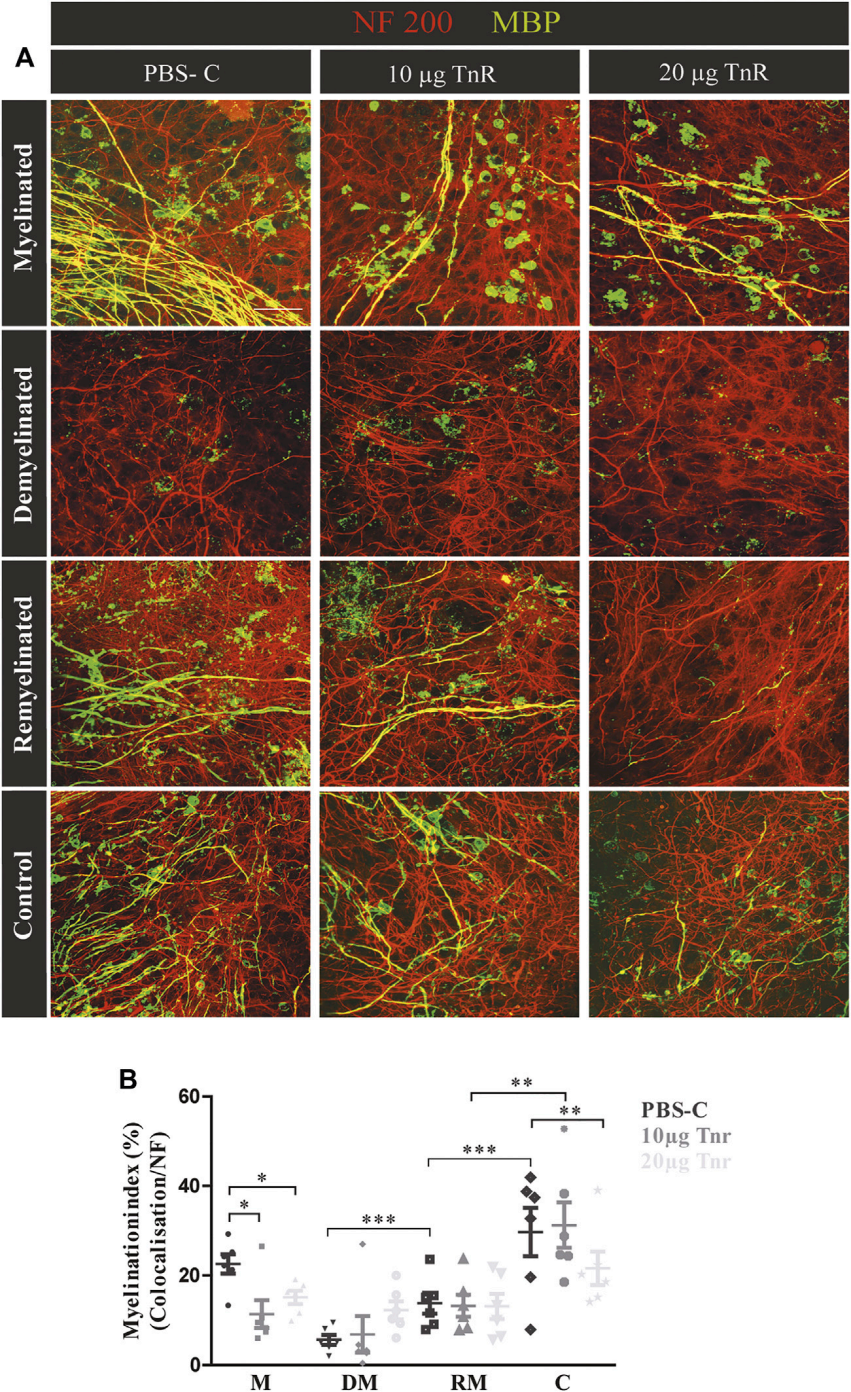
**(A,B)** Comparison of remyelination efficiency in cerebellar cultures from NMRI-mice in the presence of purified Tnr. **(A)** Labeling for neurofilament (red) and MBP (green) of live cerebellar explants kept under four different conditions: myelinated, demyelinated, remyelinated, and control. Explants were complemented either with plain PBS (control), with 10 µg, or with 20 µg immunopurified Tnr applied to the explant culture medium. MBP labeling indicates the degree of myelination under the different conditions. **(B)** The graph displays the quantification of myelination indices of the distinct conditions PBS-control (PBS-C), 10 µg Tnr, and 20 µg Tnr as applied to explant cultures kept in the different conditions (M, myelinated; DM, demyelinated; RM, remyelinated; C, control). All data are provided as mean ± SEM. Statistical significance was assessed using the two-way ANOVA (*p ≤ 0.05, **p ≤ 0.01, ***p ≤ 0.001) and Tukey’s multiple-comparisons test. Scale bars: 50 µm. Five independent experiments (N = 5) were carried out, and three (*n* = 3) explants per experiment were analyzed for each condition and each slice culture group.

Earlier studies had revealed that Tnc and Tnr regulate the differentiation of oligodendrocytes *in vitro* (Czopka et al., 2009b; Czopka et al., 2010). In order to examine whether this is also the case in a more complex setting closer to the *in vivo* situation, we studied their impact on de- and remyelination in a live cerebellar explant system (Zhang et al., 2011) (Figure 1). To this end, remyelination in cerebellar slice cultures of *Tnc* ^*+/+*^, *Tnc* ^*−/−*^, *Tnr* ^*+/+*^, and *Tnr* ^*−/−*^ mice was studied. Immunohistochemical staining with NF200 and MBP was performed in four conditions, namely, myelinated (M), demyelinated (DM), remyelinated (RM), and control (C) (Figures 2–5). The degree of myelination was assessed according to MBP-labeling along neurofilament-positive axons and developed during the first 10 days of cultivation (M, Figure 2). Upon application of lysolecithin after 10 days of cultivation, a drastic demyelination (DM) was obvious and could be observed in all genotypes studied, where MBP-staining appeared highly reduced (Figure 2) (Ulc et al., 2019). Upon withdrawal of lysolecithin, an initial remyelination (RM) was observable in both genotypes that progressed over 14 days, the endpoint of the experiment. Progressively myelinated axons were visible in the control condition (C), explants that were kept unperturbed over 25 days. To investigate the difference between *Tnc* ^*+/+*^ and *Tnc* ^*−/−*^ slices, we performed a quantification of the myelination indices. After 10 days, the *Tnc* ^*−/−*^ explants presented a higher percentage of myelinated axons compared to the wildtype that proved statistically significant (M, *Tnc* ^+/+^: 18.56% ± 7.33%, *Tnc* ^−/−^: 24.56% ± 7.9%, *p* = 0.003). Demyelination was successful (Figures 2A,B), presenting the lowest myelination grade for both genotypes (DM, *Tnc* ^*+/+*^: 2.64% ± 1.64%, *Tnc* ^*−/−*^: 3.51% ± 2.50%). Successful remyelination occurred in both genotypes, with an apparent advantage in *Tnc* ^*−/−*^ compared with *Tnc* ^*+/+*^ cultures that did not, however, achieve statistical significance (RM, *Tnc* ^*+/+*^: 24.37% ± 9.97%, *Tnc* ^*−/−*^: 31.71% ± 7.34%). The control condition (C) that had not been exposed to lysolecithin revealed an ongoing increase of myelination compared to the demyelinated condition. Interestingly, myelination in the absence of Tnc appeared comparable to the WT (C, *Tnc* ^*+/+*^: 26.80% ± 5.95%, *Tnc* ^*−/−*^: 29.35% ± 9.79%). A change in the degree of myelin formation could result from the reduced availability of axons as a consequence of fasciculation. To exclude the influence of axon fasciculation on the myelination degree, we also measured the percentage of NF200-positive fluorescence in the explants. As our results demonstrated, axon signals were only significantly increased during myelination in *Tnc* ^*−/−*^ tissue (M, *Tnc* ^+/+^: 19.83%, *Tnc* ^*−/−*^: 40.59%, *p* = 0.0013) (Supplementary Figure S2B). No significant differences considering the axon signals were detectable in the control condition (C, *Tnc* ^+/+^: 41.3%, *Tnc* ^*−/−*^: 45.3%), or during demyelination (DM, *Tnc* ^+/+^: 30.6%, *Tnc* ^*−/−*^: 45.98%). In the latter case, a large scatter of values obliterated the significance of the seeming difference (Supplementary Figure S2B). Moreover, during the successful remyelination, there were still no significant differences considering the axon signals detectable (RM, *Tnc* ^+/+^: 40.08%, *Tnc* ^*−/−*^: 43.81%). Therefore, the availability of axon surfaces for ensheathment by myelin was comparable in the different experimental samples.

### Purified Tnc Interferes With Remyelination in the Cerebellar Explant Assay

The data seemed to indicate a weak but visible retarding effect of Tnc on myelination, whereas demyelination per se was not modified by the absence of Tnc. To test this interpretation, demyelinated WT explants were directly exposed to purified Tnc isolated from the postnatal CNS (Figure 3). After lysolecithin withdrawal, 50 μg/ml Tnc was added in soluble form to the slice cultures and cultivated for 14 days (Figure 3A). This concentration range had proven efficient for stimulating cells with soluble Tnc in previous studies (Haage et al., 2019). Myelination indices were evaluated for untreated and treated remyelination and control groups (Figure 3B). No differences from the untreated group were measured by adding Tnc to the control group (C, w/o Tnc: 20.13% ± 2.66%, +Tnc: 18.53% ± 12.26%) (Figures 3A-B, A-B'). However, remyelination efficiency was significantly decreased when Tnc was added to the remyelination group in comparison with the untreated remyelination group (RM, w/o Tnc: 17.82% ± 1.61%, +Tnc: 11.15% ± 1.01%, *p* = 0.0029) (Figures 3A-A, A-A'). In the presence of Tnc, the remyelination appeared strongly impeded (Figure 3B).

### Accelerated Remyelination in Cerebellar Slice Cultures of *Tnr* ^*−/−*^ Mice

The inhibitory effects of Tnc on remyelination were in agreement with studies showing that Tnc reduces oligodendrocyte membrane extension *in vitro*. The paralogue Tnr is structurally related to Tnc and had analogous effects in that cell biological assay (Czopka et al., 2009b). Therefore, we examined the consequences of *Tnr* gene ablation in the cerebellar remyelination assay (Figure 1). To assess the difference between *Tnr* ^*+/+*^ and *Tnr* ^*−/−*^ explants (Figure 4A), we performed a quantification of the myelination indices. Here, the myelinated condition represented a slightly higher, although not statistically significant percentage of myelinated axons compared to the control group (M, *Tnr* ^*+/+*^: 23.20% ± 9.71%, *Tnr* ^*−/−*^: 24.66% ± 11.21%; Figure 4). This was not observed in the untreated control explants (*Tnr* ^*+/+*^: 16.11% ± 3.68%, *Tnr* ^*−/−*^: 15.58% ± 4.41%). As reflected in the myelination indices, demyelination by lysolecithin was successful (Figure 4B) and presented the lowest myelination grade in the demyelinated condition for both genotypes (DM, *Tnr* ^*+/+*^: 5.60 %± 1.5%, *Tnr* ^*−/−*^: 4.92% ± 2.12%). Moreover, a successful remyelination could be observed which, remarkably, appeared stronger in *Tnr* ^*−/−*^ than *Tnr* ^*+/+*^ cultures (RM, *Tnr* ^*+/+*^: 9.11% ± 3.2%, *Tnr* ^*−/−*^: 15.21% ± 6.1%, *p* = 0.0165). This observation suggested a clear inhibitory effect of *Tnr* on the remyelination process. Furthermore, we have analyzed the fraction of axon-dependent fluorescence in the different conditions, and as our results demonstrated, the intensity of NF200-positive signals in *Tnr* ^*−/−*^ mice was significantly increased in the demyelination condition (DM, *Tnr* ^*+/+*^: 29.83%, *Tnr* ^*−/−*^: 45.09%, *p* = 0.0003) (Supplementary Figure S2C). Under untreated control condition, no significant differences considering the axon signals in both genotypes were detectable (C, *Tnr* ^*+/+*^: 44.19%, *Tnr* ^*−/−*^: 54.54%). Moreover, the results showed that Tnr seemed to exert no influence on axon signals under myelination (M, *Tnr* ^*+/+*^: 45.35%, *Tnr* ^*−/−*^: 45.53%) and remyelination conditions (RM, *Tnr* ^*+/+*^: 40.08%, *Tnr* ^*−/−*^: 43.91%). In view of the apparent inhibitory effect, we tested the purified Tnr protein directly in the explant remyelination assay.

### Purified Tnr Reduces Remyelination in the Cerebellar Explant Assay

Cerebellar explants were kept in culture for 10 days, demyelinated, and subsequently remyelinated as indicated (Figure 1). To assess the impact on myelination, Tnr was added at 10 μg/ml or 20 μg/ml purified protein to the culture medium, while plain PBS instead of ECM protein was used as the control (Figure 5A). During the first 10 days of cultivation, quantification revealed a higher percentage of myelinated axons in the PBS-control compared to Tnr-treated groups (M; PBS-C: 23.07% ± 8.42%, 10 µg Tnr: 11.37% ± 3.09%, *p* = 0.0142, 20 µg Tnr: 15.12% ± 1.47%, *p* = 0.0176). Explants kept in the presence of 10 µg Tnr represented the least degree of myelination of axons, and this apparent retardation of myelination during the initial cultivation phase was highly significant (Figure 5). Demyelination upon application of lysolecithin was successful, as evidenced by a reduction of MBP-staining (DM; PBS-C: 5.16 ± 2.34%, 10 µg Tnr: 6.50% ± 3.69%, 20 µg Tnr: 9.56% ± 2.51%). Remyelination could be observed but differed between these three groups. The highest percentage of remyelinated axons was obtained in the PBS-control situation, whereas the least percentage was recorded for explants confronted with 20 μg/ml Tnr. Interestingly, in that latter situation, the myelination index was nearly the same for the remyelinated condition compared to the demyelinated condition (RM; PBS-C: 20.45% ± 7.26%, 10 µg Tnr: 13.03% ± 2.29%, 20 µg Tnr: 9.97% ± 3.30%). As a further support of Tnr-dependent inhibition, the PBS-control displayed a significant increase of remyelination compared to demyelination. In the control condition for all groups, the highest percentage of myelinated axons was noted when the explants were exposed neither to lysolecithin nor to Tnr protein (C; PBS-C: 25.75% ± 8.35%, 10 μg/ml Tnr: 23.84% ± 7.09%, 20 μg/ml Tnr: 20.37% ± 4.80%, *p* = 0.0053). Overall, these observations clearly suggest that Tnr protein reduced myelination in the cerebellar explant assay. Considering the remyelination condition, it is noteworthy that the myelination indices in the presence of Tnr did not differ from the demyelination condition, while a significantly recovery could be detected when the explants were treated with plain PBS (Figure 5B). We interpret this result as indicating that Tnr delayed recovery of myelin to some extent by maintaining the demyelination level (Figure 5B).

### LFB–PAS-Staining and MBP-Staining Reveal Successful Demyelination and Remyelination in the Different Genotypes in the Cuprizone Model *In Vivo*

**FIGURE 6:**
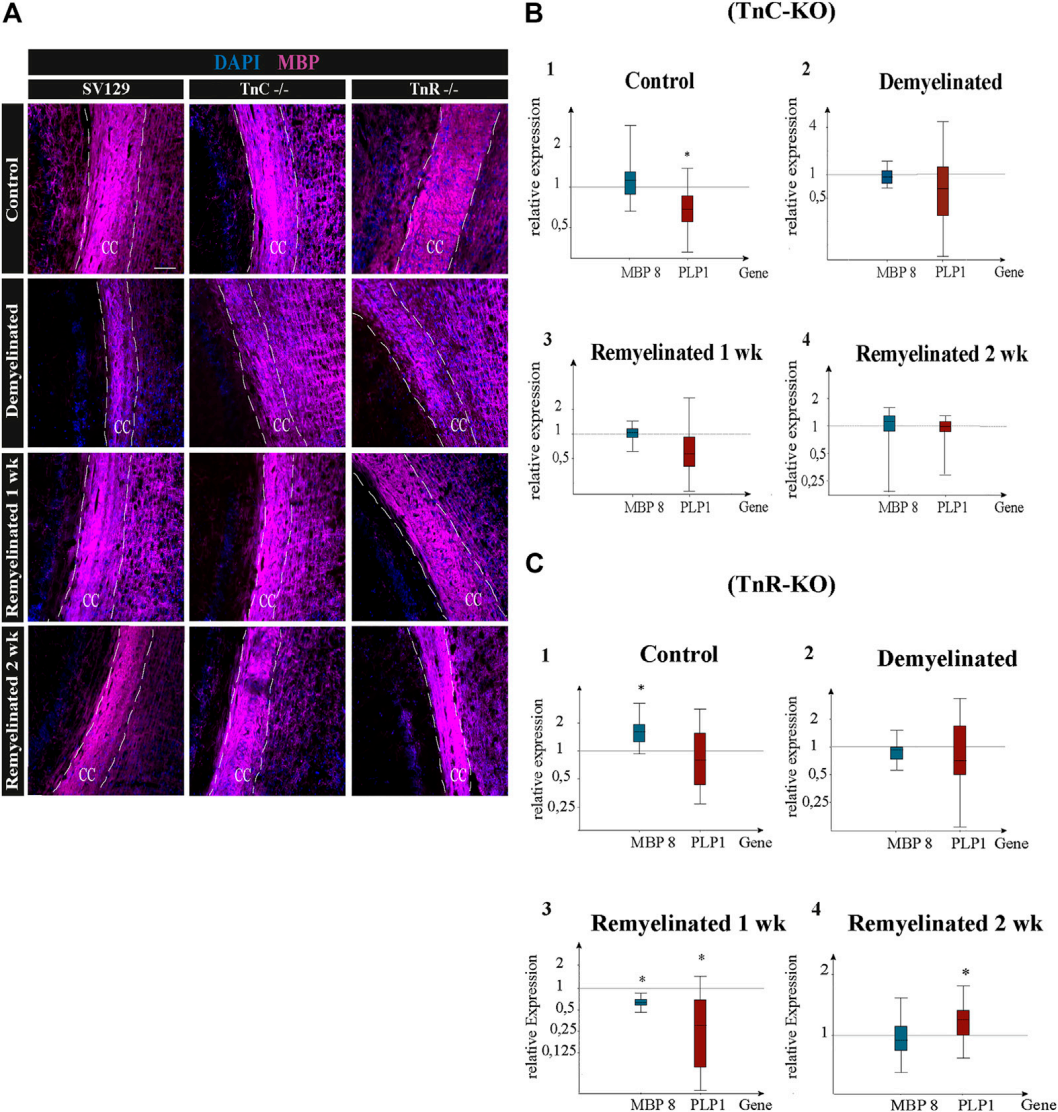
**(A–C)** Immunolabeling for MBP and gene expression analysis of the myelin genes PLP1 and MBP8 in the corpus callosum of SV129, *Tnc* ^*−/−*^, and *Tnr* ^*−/−*^ mice under control, demyelinated, and remyelinated conditions. **(A)** Immunohistochemistry with antibodies against MBP (violet) for visualizing the myelination grade in the corpus callosum of SV129, *Tnc* ^*−/−*^, and *Tnr* ^*−/−*^ mice. Four conditions were compared: control, one-week demyelinated (1 wk DM), one-week remyelinated (1 wk RM), and two-weeks remyelinated (2 wk RM). DAPI-staining (blue) shows the cell nuclei in the field of vision. The immunohistochemical staining illustrates an increased intensity of MBP in the area of the corpus callosum in the control condition for the three genotypes. In comparison with the untreated control, MBP-staining of the demyelinated condition was reduced in all genotypes, revealing efficient demyelination by cuprizone. The different genotypes behaved similarly in all four conditions. Scale bars: 100 µm. Using qPCR **(B)**, the expression analysis of the myelin genes MBP and PLP1 was carried out in corpus callosum samples of *Tnc* ^*−/−*^ and SV129 mice in “control,” “demyelinated,” “one-week remyelinated (1 wk),” and “two-weeks remyelinated (2 wk)” conditions and was represented graphically with box plots. The expression of MBP and PLP1 was normalized with reference to the control genes RPLP0 and β-actin. The expression of myelin genes in SV129 mice was set as 1. The control condition exhibited nearly unchanged expression of MBP but significantly reduced expression of PLP1 in *Tnc* ^*−/−*^ samples compared to the SV129 wildtype. The demyelinated condition revealed a downregulation of both myelin genes in *Tnc* ^*−/−*^ in comparison with SV129 samples. After one week of remyelination, PLP1 was also downregulated in *Tnc* ^*−/−*^ samples, while MBP expression remained unchanged. After two weeks of remyelination, both genotypes did not differ. **(C)** The expression of both myelin genes in *Tnr* ^*−/−*^ and SV129 mice was normalized with reference to the control genes RPLP0 and β-actin. The analysis of all data and statistical significance were assessed using LightCycler^®^96 and Rest 2009 (*p ≤ 0.05, **p ≤ 0.01, ***p ≤ 0.001). At least three animals were included in each group (N = 3) and genotype (*n* = 500 cells).

The outcome of the studies with cerebellar explants strongly suggested an inhibitory role of Tnc and Tnr in remyelination *in vitro*. This prompted the question whether these glial-derived ECM constituents interfere with the remyelination process *in vivo*. In order to study this issue, demyelination was induced in wildtype and transgenic mice by administration of a cuprizone-rich diet over a period of 6 weeks, following an established protocol (Ulc et al., 2019). To assess the myelination grade in the corpus callosum of SV129, *Tnc* ^*−/−*^, and *Tnr* ^*−/−*^ mice in the conditions “control,” “demyelination,” “one-week remyelination,” and “two-weeks remyelination,” we performed LFB–PAS- and MBP-staining of tissue sections. LFB detects phospholipids/lipoproteins in myelin, and PAS marks glycoproteins in the axons (Xiang et al., 2005; Lindner et al., 2008). In contrast, labeling for MBP focuses on the specific myelin compartment. In all genotypes, the histochemical staining revealed more pronounced myelin in the control as compared to the demyelinated condition. The reduced intensity of myelin staining in the demyelinated condition could be explained by a successful cuprizone-induced demyelination. However, some residual LFB- and MBP-staining was still visible in demyelinated tissue, although demyelination had occurred. Additionally, LFB-staining revealed a reduction of myelin also outside the corpus callosum (Supplementary Figure S1B), which suggested effective demyelination in the telencephalon. After one week of remyelination, MBP-staining increased compared to the demyelinated condition (Figure 6A). In contrast, LFB-staining did not detect differences between one-week remyelination and the demyelinated condition. Interestingly, brain sections of *Tnc* ^*−/−*^ and *Tnr* ^*−/−*^ mice displayed a higher intensity of MBP-labeling after one week of remyelination compared to the wildtype SV129 sections. The histochemical staining intensity increased further after two weeks, clearly suggestive of remyelination. According to histochemistry and to immunohistochemical analysis for MBP, we established successfully the cuprizone model in *Tnc* ^*−/−*^ and *Tnr* ^*−/−*^ mutant mice. Because histological studies relying on LFB–PAS- and MBP-staining did not allow for precise quantification, the expression of two important myelin genes was studied on the mRNA level.

### Monitoring Cuprizone-Dependent Myelin Remodeling With Oligodendrocyte Markers

Quantitative PCR was used to analyze the expression of the myelin-specific genes *MBP8* and *PLP1* from the corpus callosum of *Tnc* ^*−/−*^ and *Tnr* ^*−/−*^ mice in the conditions “control,” “demyelinated,” “one-week remyelinated,” and “two weeks remyelinated” (Figure 6). The gene expression of *MBP8* and *PLP1* was normalized to the gene expression of the housekeeping genes *RPLP0* and *β-actin* (Figures 6B,C). The expression of *MBP8* (relative expression: 1.24) in *Tnc* ^*−/−*^ compared to SV129 samples did not change and was nearly the same in each condition (Figure 6B). For *PLP1*, on the contrary, the control (Figures 6A,B) was significantly reduced in the corpus callosum of *Tnc* ^*−/−*^ compared to SV129 mice (*p* = 0.017). In the demyelination condition, a downregulation for both myelin genes was detectable. However, there were hardly any differences between *Tnc* ^*−/−*^ and SV129 mice (relative expression of *MBP8*: 0.952, *PLP1*: 0.679). After one week of remyelination, only a higher expression of *MBP8* (1.051) could be observed (Figures 6B,C), whereas *PLP1* was downregulated in *Tnc* ^*−/−*^ compared to SV129 samples (relative expression: 0.626). In comparison with SV129, after two weeks of remyelination, there were no significant differences in myelination-specific expression in the *Tnc* ^*−/−*^ samples (Figures 6B–D). In comparison with the SV129 wildtype, a significant upregulation of *MBP8* in the control condition of the *Tnr* ^*−/−*^ mice could be noted. The *PLP1* gene, on the contrary, was weakly downregulated (relative expression of PLP1: 0.80). The demyelinated condition also showed a reduced expression for *MBP8*, whereas *PLP1* was weakly downregulated (relative expression of *MBP8*: 0.910, *PLP1*: 0.812). In the condition “one-week remyelinated,” the myelin genes *MBP8* and *PLP1* in the corpus callosum of *Tnr* ^*−/−*^ mice were significantly downregulated (Figure 6C) compared to SV129 (*MBP8*, *p* = 0.001; *PLP1*, *p* = 0.004). The comparison of the two myelin genes in the *Tnr* ^*−/−*^ genotype yielded a higher expression for *MBP8* compared to *PLP1* (relative expression of *MBP8*: 0.611, *PLP1*: 0.257). After two weeks of remyelination, the expression of *MBP8* in *Tnr* ^*−/−*^ samples appeared reduced (Figures 6C,D) compared to the SV129 samples (relative expression: 0.936). For *PLP1*, on the contrary, after two weeks of remyelination, a significantly increased expression (relative expression: 1.183) could be observed in *Tnr* ^*−/−*^ samples compared to SV129 samples (*p* = 0.036). Overall, for the myelinating gene *PLP1*, in the control condition and after one and two weeks of remyelination, a higher expression in all genotypes compared to *MBP8* was evident.

### Comparison of OPCs and Mature Oligodendrocytes in the Cuprizone Model

**FIGURE 7:**
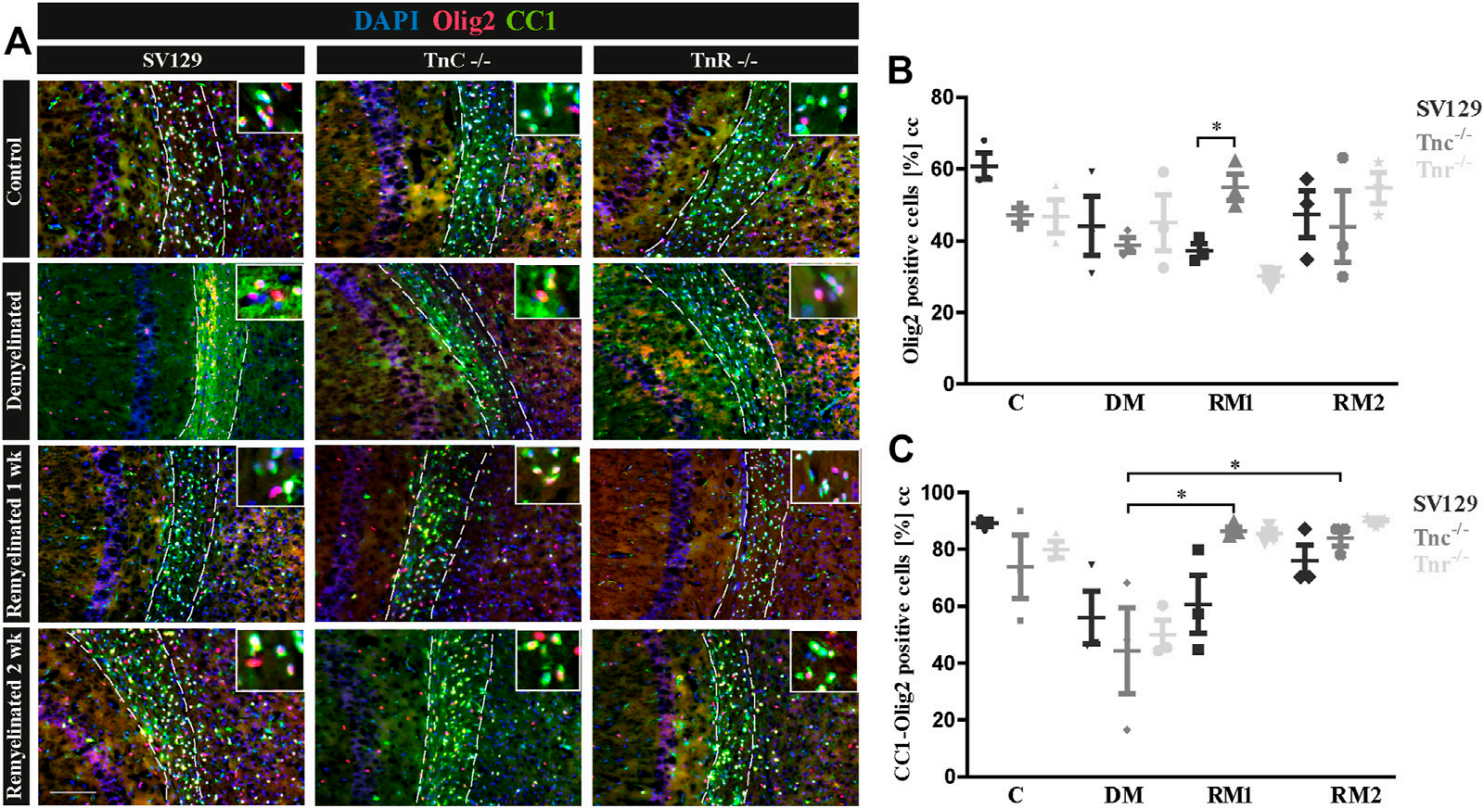
**(A–C)** Comparison of Olig2/CC1-double immunolabeling in the corpus callosum of SV129, *Tnc* ^*−/−*^, and *Tnr* ^*−/−*^ mice under control, demyelinated, and remyelinated conditions. **(A)** Using immunohistochemical labeling with antibodies against Olig2 (detects all oligodendroglia) and CC1 (detects mature oligodendrocytes), the percentage of mature oligodendrocytes in the corpus callosum of *SV129*, *Tnc* ^*−/−*^, and *Tnr* ^*−/−*^ mice was compared. The four conditions “control,” “demyelinated,” “one-week remyelinated,” and “two-weeks remyelinated” were examined. DAPI-staining marks cell nuclei in blue color. Olig2 is nucleus-based staining, and CC1 is located in the cytosol. In the absence of treatment, a strong labeling of CC1-positive cells (green) was detectable in the corpus callosum (CC) of all genotypes. Mature CC1/Olig2-positive cells were reduced in the demyelinated condition. **(B)** Quantification of Olig2-positive cells in the particular conditions (C: control, DM: demyelinated, RM1: one-week remyelinated, RM2: two-weeks remyelinated). **(C)** Quantification of CC1/Olig2-double immunopositive cells. The quantification confirms a lower percentage of mature oligodendrocytes in the demyelinated condition compared to the control for all genotypes. All data are provided as mean ± SEM. The statistical significance was assessed by using the two-way ANOVA (*p ≤ 0.05, **p ≤ 0.01, ***p ≤ 0.001) and Tukey’s multiple-comparisons test. The micrograph of the illustration was captured via Axiophot. Scale bars: illustration: 100 µm detail, pictures: 50 μm; at least *N* = 3 animals were used for each group and genotype, and at least *n* = 500 cells were evaluated for each individual animal.

To gain insight into the oligodendrocyte lineage, studies using the markers Olig2 and CC1 were performed (Figure 7). For an individual quantification of mature oligodendrocytes, we carried out Olig2/CC1 double immunostaining to investigate the differentiation process of OPCs dependent on tenascins. Immunopositive cells were counted comparatively in SV129, *Tnc* ^*−/−*^, and *Tnr* ^*−/−*^ mice. In the demyelinated condition, we observed (Figure 7C) cells that were Olig2/CC1-positive (demyelination; SV129: 56.10% ± 13.08%, *Tnc* ^*−/−*^: 44.33% ± 21.27%, *Tnr* ^*−/−*^: 50.02% ± 7.27%) compared to the control condition (SV129: 89.31% ± 1.92%, *Tnc* ^*−/−*^: 73.8% ± 15.75%, *Tnr* ^*−/−*^: 79.92% ± 4.11%). The quantitative analysis of Olig2-positive cells confirmed the presence of oligodendroglia in the demyelinated condition for all genotypes (Figure 7B). A complete elimination of oligodendroglia, however, could not be detected upon demyelination. The knockout of both tenascins did not substantially modify Olig2- or Olig2/CC1-positive populations in the untreated control condition (C; Figures 7B,C). Olig2- and Olig2/CC1-positive OPCs and oligodendrocytes, respectively, were monitored upon demyelination and after one and two weeks of remyelination. Possibly due to the variability of the system, no statistical significance could be achieved in most of the comparisons (Figures 7A,B). To accentuate the effects, a more extended exposure to cuprizone might be necessary. Interestingly, after one week of remyelination, however, in comparison with the demyelinated condition, *Tnc* ^*−/−*^ sections presented a significant increase of Olig2-positive cells (Figure 7B) and a significant increase of mature oligodendrocytes (*Tnc* ^*−/−*^: 86.41 % ± 2.10%, *p* = 0.0194) (Figure 7C). After two weeks of remyelination, the significant increase of Olig2/CC1-positive cells compared to the demyelinated condition persisted in the *Tnc* ^*−/−*^ knockout (RM2; *Tnc* ^*−/−*^: 84.13% ± 4.24%, *p* = 0.0330). This might reflect the inhibitory effect of Tnc on OPC migration and maturation, so that, in the absence of Tnc, the immigration of OPCs is favored. This is in agreement with an earlier report showing enhanced invasion of OPCs into the *Tnc* ^*−/−*^ optic nerve (Garcion et al., 2001).

### Expression of the DSD-1/Phosphacan Proteoglycan in the Cuprizone Model

**FIGURE 8:**
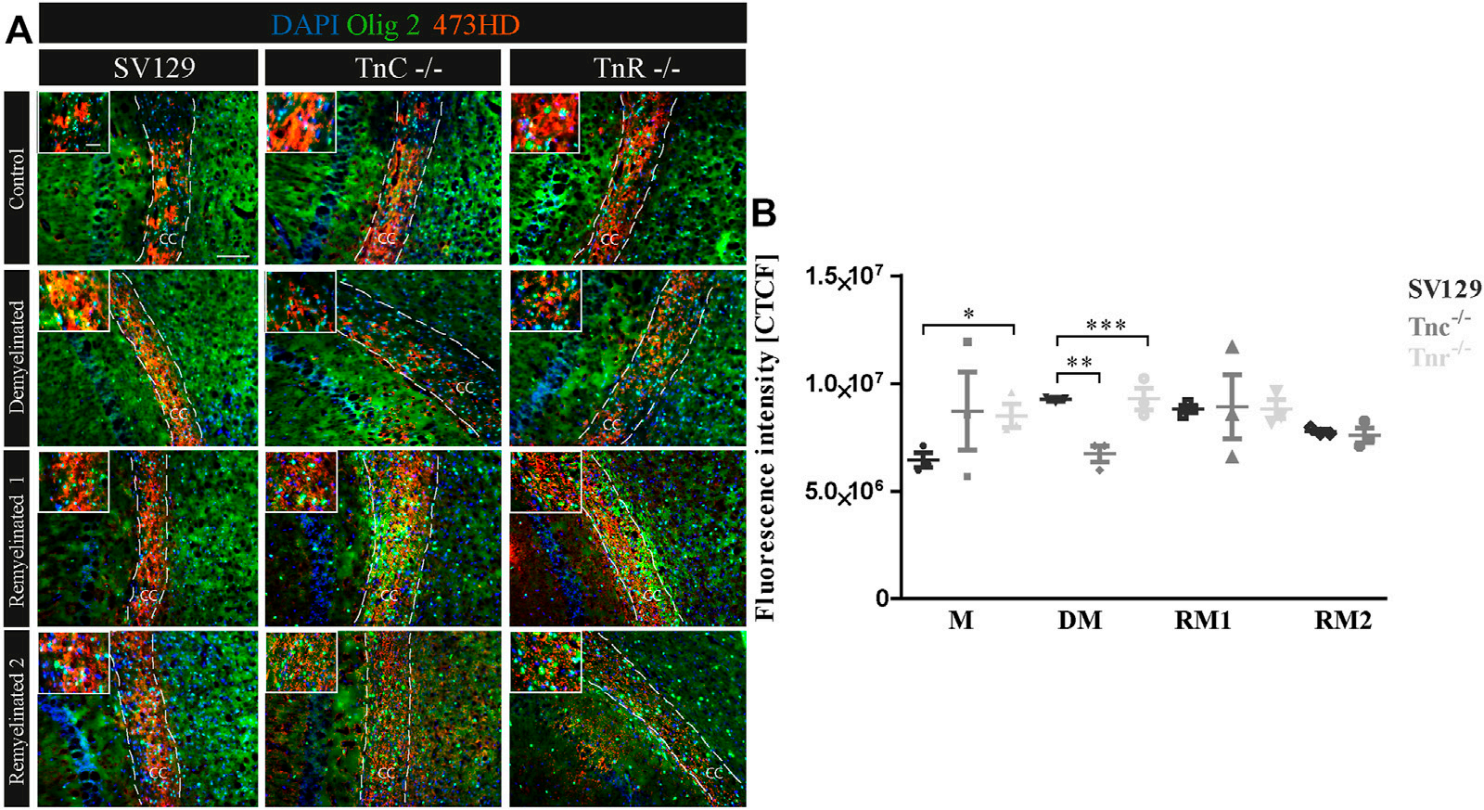
**(A,B)** Comparative analysis of Olig2/473HD-staining in the corpus callosum of SV129, *Tnc* ^*−/−*^, and *Tnr* ^*−/−*^ mice under control, demyelinated, and remyelinated conditions. Immunohistochemical labeling **(A)** with antibodies against Olig2 (detects all oligodendroglia) and 473HD (detects chondroitin sulfate) of SV129, *Tnc* ^*−/−*^, and *Tnr* ^*−/−*^ brain sections under different conditions (control, demyelinated, one-week remyelinated, two-weeks remyelinated) is shown. **(B)** Quantitative analysis of the 473HD fluorescence intensity (CTCF) in the corpus callosum above the region of the hippocampus. The fluorescence intensity was significantly reduced within the corpus callosum of *Tnr* ^*−/−*^ in control and demyelinated conditions. DAPI stains cell nuclei in blue. Scale bar: illustration: 100 μm, detail pictures: 50 µm. *N* = 3 animals were used for each group and genotype, and at least *n* = 500 cells were evaluated for each individual animal.

A previous study had shown that chondroitin sulfate regulates the proliferative maintenance of OPCs and inhibits further differentiation toward mature oligodendrocytes (Karus et al., 2016). In order to trace a potential inhibitory influence of chondroitin sulfate, we performed immunohistochemical staining of brain sections of SV129, *Tnc* ^*−/−*^, and *Tnr* ^*−/−*^ mice using the markers Olig2 and 473HD. The mAb 473HD reacts with the particular DSD-1-chondroitin sulfate epitope (Faissner et al., 1994a; Clement et al., 1998). To measure the expression level, the corrected total cell fluorescence (CTCF) of the 473HD-positive area in the corpus callosum was determined. In the absence of treatment (Figure 8), immunostaining revealed a higher intensity of 473HD in *Tnc* ^*−/−*^ and *Tnr* ^*−/−*^ sections compared to wildtype SV129 (C; SV129: 6442954.67 ± 591709.4, *Tnc* ^−/-^: 8716938.67 ± 3131031.83, *Tnr* ^*−/−*^: 8495473.33 ± 946020.84, *p* = 0.0333). In contrast, in the demyelinated condition, *Tnc* ^*−/−*^ and *Tnr* ^*−/−*^ sections presented a significantly reduced 473HD-staining and fluorescent intensity than those observed in the SV129 WT (DM; SV129: 9264336 ± 149233.09, *Tnc* ^−/−^: 6727202.67 ± 656442.63, *p* = 0.0028, *Tnr* ^−/−^: 8148442.67 ± 164110.66, *p* = 0.0010). Under remyelinated conditions, the intensity of immunostaining in SV129 WT seemed slightly elevated in comparison with that in *Tnc* ^*−/−*^ and *Tnr* ^*−/−*^. However, this could not be reflected in measurements of the fluorescence intensity (RM1; SV129: 8811688 ± 284248, *Tnc* ^*−/−*^: 8906437.33 ± 2579201.47, *Tnr* ^*−/−*^: 9285434.67 ± 868391.98). After two weeks of remyelination, no differences between the genotypes could be detected (RM2; SV129: 7769445.33 ± 164110.66, *Tnc* ^*−/−*^: 10327677.33 ± 5335489.68, *Tnr* ^*−/−*^: 8811688 ± 752049.52). Relating to an increased 473HD-staining and measured fluorescent intensity in *Tnr* ^*−/−*^ sections under control condition, these results suggest that Tnr has an inhibitory influence on chondroitin sulfates in the absence of treatment. These results are consistent with an inhibitory influence of chondroitin sulfate on oligodendrocyte differentiation. However, in the demyelinated condition, the fluorescence intensity of 473HD in both *Tnc* ^*−/−*^ and *Tnr* ^*−/−*^ mice was significantly decreased, which suggests that both tenascins at least in the inflammatory environment are essential for the normal expression of oligodendrocyte precursors.

## Discussion

In the present study, we investigated the influence of the glycoproteins Tnc and especially Tnr on the differentiation and remyelination efficiency of oligodendrocytes. Previous studies of the laboratory had revealed that the Tnc protein of the tenascin family of EM glycoproteins exerts inhibitory effects on the differentiation of oligodendrocyte precursor cells *in vitro* (Kiernan et al., 1996; Czopka et al., 2009b). While Tnc is expressed by neural stem cells and astrocyte precursors and downregulated postnatally, Tnr is upregulated during the postnatal period and clearly expressed by a subpopulation of neurons and maturing oligodendrocytes (Czopka et al., 2009b; Faissner et al., 2017). When comparing the *Tnc* ^*+/+*^ and *Tnc* ^*−/−*^ explant cultures, the highest degree of myelination was observed in the untreated situation. Overall, the *Tnc* ^*−/−*^ explants seem to acquire a higher degree of myelination. The addition of lysolecithin led to a strong demyelination, and successful remyelination was achieved upon removal of the compound. In all conditions tested, more myelin was observable in *Tnc* ^*−/−*^ explants. Tnc inhibits the migration and differentiation of OPCs after demyelination and thereby reduces the remyelination of demyelinated axons. Alternatively, a Tnc-dependent activation of astrocytes may result in reduced myelination of axons (Nash et al., 2011). Tnc accumulation in demyelinated plaques thus might prevent successful remyelination (Gutowski et al., 1999; Czopka et al., 2009b; Zhao et al., 2009). The comparison of *Tnr* ^*+/+*^ and *Tnr* ^*−/−*^ explant cultures displayed the highest degree of myelination in the untreated situation. In particular, the *Tnr* ^*−/−*^ explants displayed the most extensive myelination. Upon demyelination by addition of lysolecithin, a strong reduction of myelin could be obtained. Compared to normal development, newly formed myelin membranes are thinner upon remyelination (Dubois-Dalcq et al., 2008). After demyelination, a strong increase in the proliferation rate of OPCs ensues in the explants. The OPCs subsequently differentiate into mature oligodendrocytes and myelinate the demyelinated axons (Zhang et al., 2011). Altogether, the *Tnr* ^*−/−*^ explants were characterized by a higher degree of myelination and remyelination than the *Tnr* ^*+/+*^ wildtype. By comparing the several conditions from each genotype in view of myelinated and control conditions, it is noticeable that the myelination index in the control condition is higher than that in the myelinated condition. This is not what one firstly expected because a longer cultivation time might favor ongoing myelination. However, in organotypic slice culture, a timing of myelin onset in white matter lesions was reported, and it was shown that the number of MBP-positive oligodendrocytes was after 9 div higher than after 13 div (Dean et al., 2011). This is consistent with studies reporting that the highest degree of myelination can be observed after 12 div (Zhang et al., 2011; Shen and Yuen, 2020).

As previously reported, the knockout of *Tnc* leads to better myelination and remyelination rates through an interplay of increased migration and differentiation rates (Czopka et al., 2009b; Zhao et al., 2009; Zhang et al., 2011). The analogous inhibitory effect of Tnr on the formation of new myelin membranes and remyelination can be explained by structural similarities between Tnr and Tnc as members of the tenascin gene family. Interestingly, both Tnc and Tnr interfere with the formation of myelin membranes *in vitro* (Czopka et al., 2009b). The inhibitory property of Tnr could explain the comparatively faster remyelination observed in *Tnr* ^*−/−*^ explants. The inhibitory effect of Tnc on OPC migration could be ascribed to FNIII domains 7 and 8 (Kiernan et al., 1996). The corresponding domain responsible in Tnr has not been localized yet. However, previous studies have revealed that both Tnc and Tnr interfere with the activation of RhoA GTPase, which results in an impaired formation of myelin membranes (Wenk et al., 2000; Czopka et al., 2009b). Accordingly, it could be expected that the deletion of Tnr or Tnc unleashes the RhoA GTPase which has a positive effect on the formation of myelin membranes. Along these lines, thicker and more extensive myelin membranes were generated in *Tnr* ^*−/−*^ in comparison with wildtype mice. The myelination process can be divided into two phases: first, the extension of oligodendrocyte processes toward axons and, subsequently, the wrapping of axons with several layers of myelin membranes (Zuchero et al., 2015). Myelin basic protein (MBP) activates actin-degrading proteins such as cofilin and gelsolin, which promotes actin degradation and thereby may support the process of myelin wrapping (Zuchero et al., 2015). However, in the explant paradigm, the expression of MBP did not significantly vary between the wildtype, *Tnc* ^*−/−*^, and *Tnr* ^*−/−*^ tissues.

It is known that ECM components are upregulated after CNS injury, which leads to the formation of a glial scar resulting in an impairment of oligodendrocyte differentiation and remyelination (Lau et al., 2012; Jakovcevski et al., 2013; Deng et al., 2015; Roll and Faissner, 2019; Ghorbani and Yong, 2021). In *ex vivo* slices exposed to purified Tnc protein, the remyelination efficiency was significantly impaired. This is in line with previous studies showing that cultivation of OPCs on a Tnc substrate leads to an impaired differentiation toward MBP-positive oligodendrocytes effected via a reduced Fyn activity. This effect seems to be mediated by contactin-1 (Cntn1) whose activation results not only in a reduced Fyn activity but also in a tampered Sam68 expression. Both genes are required for MBP expression and oligodendrocyte differentiation (Czopka et al., 2010). Treatment of cerebellar explants with 20 μg/ml purified Tnr protein, on the contrary, confined remyelination to the level of the demyelinated situation. This was to be expected because remyelination was more effective in *Tnr* ^*−/−*^ compared to *Tnr* ^*+/+*^ explants and Tnr was known to tamper the formation of myelin membranes (Czopka et al., 2009b). Accordingly, when plain PBS was added to the explants, the interference with remyelination vanished. A straightforward explanation would be that the RhoA-signaling pathway is inhibited by the Tnr protein (Czopka et al., 2009b).

So far, four types of receptors have been identified for Tnc: cell adhesion molecules of the Ig-superfamily, integrins, heparan sulfate proteoglycans, and receptor tyrosine phosphatases (Czopka et al., 2010; Tucker and Chiquet-Ehrismann, 2015). Based on the structural resemblance between Tnr and Tnc, the aforementioned receptors are also conceivable for the ligand Tnr. For example, the cell adhesion molecule F3/F11 (also known as Cntn1) is a neuronal receptor of Tnr and implicated in the regulation of axonal growth (Xiao et al., 1996; Zacharias et al., 2002; Zacharias and Rauch, 2006). Interestingly, F3 is expressed by oligodendrocytes and might hence serve as the Tnr receptor in this cell type as well (Koch et al., 1997).

The outcome of the *in vitro* assays prompted us to investigate potential effects of tenascins on myelin formation *in vivo*. To this end, demyelination was induced with cuprizone, and the subsequent remyelination was studied using LFB–PAS-staining and immunohistochemistry for MBP. The cuprizone model had been successfully established in C57BL/6 mice (Torkildsen et al., 2008) and could also successfully be transferred to the *Tnc* ^*−/−*^ and *Tnr* ^*−/−*^ mice analyzed in our study. Extensive myelin was documented in the corpus callosum, which was expected because myelination is extensive there (Reyes-Haro et al., 2013). Successful demyelination was achieved by cuprizone added to the chow, as revealed by attenuated LFB–PAS-staining in- and outside of the corpus callosum, consistent with previous studies (Skripuletz et al., 2008). Residual myelin proteins and lipids after demyelination were detectable using these markers (Czopka et al., 2009a), as reported by others (Lindner et al., 2008).

The analysis of sequential expression of the myelin genes PLP1 and MBP by qPCR demonstrated successful de- and remyelination in the corpus callosum of SV129 wildtype, *Tnc* ^*−/−*^, and *Tnr* ^*−/−*^ mice and confirmed the successful functionality of the cuprizone model. For all genotypes, demyelination resulted in a significantly reduced expression of PLP1. The cholesterol-associated tetraspanin PLP1 is the major protein of CNS myelin and therefore serves as a reliable indicator of cuprizone-induced demyelination. Interestingly, in the situation of demyelination, PLP1 expression in *Tnc* ^*−/−*^ appeared reduced compared to the wildtype. It must be kept in mind that the degree of demyelination in the corpus callosum fluctuates (Sachs et al., 2014). Thus, more extensive demyelination occurs in the caudal area of the corpus callosum (Steelman et al., 2012), which may explain differences of expression between SV129 and *Tnc* ^*−/−*^ knockout mice.

After one week of remyelination, the expression of MBP remained unchanged, whereas the expression of PLP1 was reduced in *Tnc* ^*−/−*^ compared to the SV129 wildtype mice. As Tnc inhibits the differentiation of OPCs toward the MBP-expressing stage in culture (Kiernan et al., 1996; Czopka et al., 2009b), one would have expected an accelerated maturation of myelin in the *Tnc* ^*−/−*^ tissue, which was not the case. However, MBP and PLP1 merely represent two of the genes involved in myelin formation, which also strongly depends on neuron–glia interactions (Nave, 2010). Furthermore, RNA and protein expression are not always closely correlated. For example, housekeeping genes possess a stable RNA–protein ratio, whereas regulatory proteins are broken down quickly. Also, post-translational modifications may generate a considerable variety of proteins (Stark et al., 2006; Guo et al., 2008; Vogel and Marcotte, 2012). Therefore, a low level of message does not necessarily signify a minor protein amount of PLP1 and MBP in the corpus callosum.

MBP accounts for 30% of the protein amount contributed by PLP1 (Lindner et al., 2008). Post-translational modification of the classic 18.5 kDa MBP may result in the isoforms C1–C8 (Zhang et al., 2012; Harauz and Boggs, 2013). The primers used for qPCR in our case detect all these isoforms. Interestingly, the isoform MBP8 is upregulated in MS patients and would merit further study using isoform-specific primers (Boggs, 2006). In the assays using *ex vivo* cerebellar explants, the recovery of MBP in demyelinated tissue was significantly retarded in the *Tnr* ^*−/−*^ specimen after one week of remyelination. This is consistent with our former observation that Tnr promotes temporal differentiation of OPCs *in vitro* (Czopka et al., 2009b).

In order to address the differentiation process of OPCs dependent on tenascin proteins *in vivo*, the tissues were studied using the markers Olig2 and CC1 in combination. Demyelination by cuprizone caused a reduced proportion of immature oligodendrocytes and oligodendroglia in all genotypes compared to the untreated control. This reflects the fact that cuprizone disrupts the mitochondrial metabolism and thereby causes the death of oligodendrocytes (Lindner et al., 2008; Skripuletz et al., 2008; Torkildsen et al., 2008; Kipp et al., 2009; Sachs et al., 2014). The degree of demyelination, however, fluctuates and can vary in the tissue (Sachs et al., 2014). Therefore, the elimination of oligodendrocytes was not complete. After one week of remyelination, a significant increase of oligodendroglia could be noted, especially in *Tnc* ^*−/−*^ brain slices. Previous studies have pointed out that Tnc has an inhibitory effect on the migration and differentiation of OPCs (Kiernan et al., 1996; Garcion et al., 2001; Garwood et al., 2004; Czopka et al., 2009b; Jakovcevski et al., 2013; Brosicke and Faissner, 2015). Along these lines, our results are consistent with the interpretation that Tnc retards the differentiation of OPCs to mature oligodendrocytes. Therefore, it is conceivable that the OPCs migrate more efficiently to the demyelinated areas after demyelination in the *Tnc* ^*−/−*^ mice than in the wildtype mice. Altogether, we had the impression that remyelination was eased in *Tnc* ^*−/−*^ and *Tnr* ^*−/−*^ compared to SV129 wildtype brain slices. This effect, however, was weak and did not attain statistical significance after the short six weeks of exposure to cuprizone. Whether the observed inhibitory properties of tenascins on remyelination become more effective in a model of chronic demyelination has to be tested in future studies. Of note, the development of EAE was tampered in the *Tnc* ^*−/−*^ mice (Momcilovic et al., 2017).

Recent studies indicated that chondroitin sulfate proteoglycans (CSPGs) maintain OPCs in a proliferative state and inhibit further differentiation into mature oligodendrocytes (Karus et al., 2016; Keough et al., 2016). We have shown previously that OPCs express the DSD-1/phosphacan CSPG epitope recognized by the monoclonal antibody 473HD and used this marker and Olig2 to investigate CSPG expression by OPCs in our model (Faissner et al., 1994b; Garwood et al., 1999; Karus et al., 2016). The DSD-1-epitope is downregulated with maturation of OPCs which would result in reduced expression in maturing tissue. The observation that the epitope is well detectable in our models suggests that, in addition to oligodendrocytes, other cell types such as astrocytes secrete CSPGs into the ECM (Maeda et al., 2010; Okuda et al., 2014). An accelerated maturation of OPCs in *Tnc* ^*−/−*^ and *Tnr* ^*−/−*^ tissues after cuprizone-induced demyelination could be explained by the observation that OPCs mature faster in the absence of tenascins. Mature oligodendrocytes no longer secrete chondroitin sulfate (Galtrey and Fawcett, 2007). Along these lines, the increased proportion of CSPG in the demyelinated SV129 wildtype could be explained by the inhibitory effect of tenascins on the differentiation of OPCs. After remyelination, a lower level of CSPG in tenascin knockouts than in SV129 wildtype mice was detectable. This observation may indicate a greater remyelination efficiency in the absence of both tenascins. This may be a consequence of an increased transition of OPCs from the proliferative to the differentiative state. The co-labeling with the markers Olig2 and CC1 confirmed that more mature oligodendrocytes were formed in the absence of Tnc and Tnr in the remyelination condition after one week than in the wildtype, where a higher amount of CSPG was observed. Remyelination is promoted by inhibiting the synthesis of CSPGs (Keough et al., 2016; Pu et al., 2018). The presence of CSPGs retards the maturation of OPCs and prevents their further differentiation (Galtrey and Fawcett, 2007; Lau et al., 2012). Interestingly, both CSPGs and tenascins are upregulated after brain injuries, in particular also in MS lesions (Dauth et al., 2016). Our observations so far are in agreement with the asserted inhibitory influence of CSPGs on OPC differentiation and remyelination. The tenascins bind to the CSPGs aggrecan, phosphacan, neurocan, and versican (Perides et al., 1993; Jakovcevski et al., 2013). The joint occurrence of CSPGs and tenascins in the ECM of MS patients as well as the reduced 473HD labeling in *Tnc* ^*−/−*^ and *Tnr* ^*−/−*^ brain sections might indicate that an interaction between the two ECM molecules could cooperate with regard to the inhibitory effect on remyelination (Ghorbani and Yong, 2021).

Taken together, this study revealed that both Tnc and Tnr intervene in the remyelination capacity of oligodendrocytes. This could be shown by studying the remyelination behavior of WT and knockout specimens, and by adding the purified ECM proteins to lysolecithin-treated WT cerebellar explants. In order to examine their roles in an *in vivo* situation, the cuprizone model was successfully established in *Tnc* ^*−/−*^ and *Tnr* ^*−/−*^ mice, as confirmed by LFB- and MBP-staining. While demyelination and remyelination occurred as expected, the differences between the WT, *Tnc* ^*−/−*^, and *Tnr* ^*−/−*^ mouse lines were small and did not achieve statistical significance with respect to several parameters. This may be due to the duration of the exposure to cuprizone, which was limited to six weeks, reflecting an acute lesion situation. The tendencies observed may become relevant in a chronic setting, where cuprizone treatment could be extended to ten weeks. The roles of tenascins in the mimic of a chronic disease course remain to be explored in a future study.

## Data Availability

The raw data supporting the conclusions of this article will be made available by the authors, without undue reservation.

## References

[B1] Barros C. S. Franco S. J. Muller U. (2011). Extracellular Matrix: Functions in the Nervous System. Cold Spring Harbor Perspect. Biol. 3 (1), a005108. 10.1101/cshperspect.a005108 PMC3003458 21123393

[B2] Bartsch S. Bartsch U. Dorries U. Faissner A. Weller A. Ekblom P. (1992). Expression of Tenascin in the Developing and Adult Cerebellar Cortex. J. Neurosci. 12 (3), 736–749. 10.1523/JNEUROSCI.12-03-00736.1992 1372043 PMC6576029

[B3] Becker T. Anliker B. Becker C. G. Taylor J. Schachner M. Meyer R. L. (2000). Tenascin-R Inhibits Regrowth of Optic Fibers *In Vitro* and Persists in the Optic Nerve of Mice after Injury. Glia 29 (4), 330–346. 10.1002/(sici)1098-1136(20000215)29:4<330::aid-glia4>3.0.co;2-l 10652443

[B4] Birgbauer E. Rao T. S. Webb M. (2004). Lysolecithin Induces Demyelination *In Vitro* in a Cerebellar Slice Culture System. J. Neurosci. Res. 78 (2), 157–166. 10.1002/jnr.20248 15378614

[B5] Boggs J. M. (2006). Myelin Basic Protein: a Multifunctional Protein. Cell. Mol. Life Sci. 63 (17), 1945–1961. 10.1007/s00018-006-6094-7 16794783 PMC11136439

[B6] Bolte S. Cordelières F. P. (2006). A Guided Tour into Subcellular Colocalization Analysis in Light Microscopy. J. Microsc. 224 (Pt 3), 213–232. 10.1111/j.1365-2818.2006.01706.x 17210054

[B7] Bradl M. Lassmann H. (2010). Oligodendrocytes: Biology and Pathology. Acta Neuropathol. 119 (1), 37–53. 10.1007/s00401-009-0601-5 19847447 PMC2799635

[B8] Brösicke N. Faissner A. (2015). Role of Tenascins in the ECM of Gliomas. Cell Adhes. Migration 9 (1-2), 131–140. 10.1080/19336918.2014.1000071 PMC4422794 25695402

[B9] Clement A. M. Nadanaka S. Masayama K. Mandl C. Sugahara K. Faissner A. (1998). The DSD-1 Carbohydrate Epitope Depends on Sulfation, Correlates with Chondroitin Sulfate D Motifs, and Is Sufficient to Promote Neurite Outgrowth. J. Biol. Chem. 273 (43), 28444–28453. 10.1074/jbc.273.43.28444 9774473

[B10] Czopka T. Hennen E. von Holst A. Faissner A. (2009a). Novel Conserved Oligodendrocyte Surface Epitope Identified by Monoclonal Antibody 4860. Cell Tissue Res 338 (2), 161–170. 10.1007/s00441-009-0868-9 19798513

[B11] Czopka T. von Holst A. ffrench-Constant C. Faissner A. (2010). Regulatory Mechanisms that Mediate Tenascin C-dependent Inhibition of Oligodendrocyte Precursor Differentiation. J. Neurosci. 30 (37), 12310–12322. 10.1523/JNEUROSCI.4957-09.2010 20844127 PMC3842490

[B12] Czopka T. Von Holst A. Schmidt G. Ffrench-Constant C. Faissner A. (2009b). Tenascin C and Tenascin R Similarly Prevent the Formation of Myelin Membranes in a RhoA-dependent Manner, but Antagonistically Regulate the Expression of Myelin Basic Protein via a Separate Pathway. Glia 57 (16), 1790–1801. 10.1002/glia.20891 19459213

[B13] Dauth S. Grevesse T. Pantazopoulos H. Campbell P. H. Maoz B. M. Berretta S. (2016). Extracellular Matrix Protein Expression Is Brain Region Dependent. J. Comp. Neurol. 524 (7), 1309–1336. 10.1002/cne.23965 26780384 PMC7714387

[B14] De Santis I. Lorenzini L. Moretti M. Martella E. Lucarelli E. Calzà L. (2021). Co-Density Distribution Maps for Advanced Molecule Colocalization and Co-distribution Analysis. Sensors 21 (19), 6385. 10.3390/s21196385 34640704 PMC8513075

[B15] Dean J. M. Riddle A. Maire J. Hansen K. D. Preston M. Barnes A. P. (2011). An Organotypic Slice Culture Model of Chronic white Matter Injury with Maturation Arrest of Oligodendrocyte Progenitors. Mol. Neurodegeneration 6, 46. 10.1186/1750-1326-6-46 PMC3163199 21729326

[B16] Deng Y.-P. Sun Y. Hu L. Li Z.-H. Xu Q.-M. Pei Y.-L. (2015). Chondroitin Sulfate Proteoglycans Impede Myelination by Oligodendrocytes after Perinatal white Matter Injury. Exp. Neurol. 269, 213–223. 10.1016/j.expneurol.2015.03.026 25862289

[B17] Dityatev A. Schachner M. Sonderegger P. (2010). The Dual Role of the Extracellular Matrix in Synaptic Plasticity and Homeostasis. Nat. Rev. Neurosci. 11 (11), 735–746. 10.1038/nrn2898 20944663

[B18] Dubois-Dalcq M. Williams A. Stadelmann C. Stankoff B. Zalc B. Lubetzki C. (2008). From Fish to Man: Understanding Endogenous Remyelination in central Nervous System Demyelinating Diseases. Brain 131 (Pt 7), 1686–1700. 10.1093/brain/awn076 18474520 PMC2516372

[B19] Faissner A. Scholze A. Götz B. (1994b). Tenascin Glycoproteins in Developing Neural Tissues: Only Decoration? Perspect. Dev. Neurobiol. 2 (1), 53–66. 7530144

[B20] Faissner A. Clement A. Lochter A. Streit A. Mandl C. Schachner M. (1994a). Isolation of a Neural Chondroitin Sulfate Proteoglycan with Neurite Outgrowth Promoting Properties. J. Cell Biol 126 (3), 783–799. 10.1083/jcb.126.3.783 7519189 PMC2120143

[B21] Faissner A. Kruse J. (1990). J1/tenascin Is a Repulsive Substrate for central Nervous System Neurons. Neuron 5 (5), 627–637. 10.1016/0896-6273(90)90217-4 1699568

[B22] Faissner A. Reinhard J. (2015). The Extracellular Matrix Compartment of Neural Stem and Glial Progenitor Cells. Glia 63 (8), 1330–1349. 10.1002/glia.22839 25913849

[B23] Faissner A. Roll L. Theocharidis U. (2017). Tenascin-C in the Matrisome of Neural Stem and Progenitor Cells. Mol. Cell Neurosci. 81, 22–31. 10.1016/j.mcn.2016.11.003 27836730

[B24] Franklin R. J. M. Ffrench-Constant C. (2017). Regenerating CNS Myelin - from Mechanisms to Experimental Medicines. Nat. Rev. Neurosci. 18 (12), 753–769. 10.1038/nrn.2017.136 29142295

[B25] Franklin R. J. M. Ffrench-Constant C. (2008). Remyelination in the CNS: from Biology to Therapy. Nat. Rev. Neurosci. 9 (11), 839–855. 10.1038/nrn2480 18931697

[B26] Galtrey C. M. Fawcett J. W. (2007). The Role of Chondroitin Sulfate Proteoglycans in Regeneration and Plasticity in the central Nervous System. Brain Res. Rev. 54 (1), 1–18. 10.1016/j.brainresrev.2006.09.006 17222456

[B27] Garcion E. Faissner A. ffrench-Constant C. (2001). Knockout Mice Reveal a Contribution of the Extracellular Matrix Molecule Tenascin-C to Neural Precursor Proliferation and Migration. Development 128 (13), 2485–2496. 10.1242/dev.128.13.2485 11493565

[B28] Garwood J. Garcion E. Dobbertin A. Heck N. Calco V. ffrench-Constant C. (2004). The Extracellular Matrix Glycoprotein Tenascin-C Is Expressed by Oligodendrocyte Precursor Cells and Required for the Regulation of Maturation Rate, Survival and Responsiveness to Platelet-Derived Growth Factor. Eur. J. Neurosci. 20 (10), 2524–2540. 10.1111/j.1460-9568.2004.03727.x 15548197

[B29] Garwood J. Schnädelbach O. Clement A. Schütte K. Bach A. Faissner A. (1999). DSD-1-proteoglycan Is the Mouse Homolog of Phosphacan and Displays Opposing Effects on Neurite Outgrowth Dependent on Neuronal Lineage. J. Neurosci. 19 (10), 3888–3899. 10.1523/JNEUROSCI.19-10-03888.1999 10234020 PMC6782734

[B30] Ghorbani S. Yong V. W. (2021). The Extracellular Matrix as Modifier of Neuroinflammation and Remyelination in Multiple Sclerosis. Brain 144, 1958–1973. 10.1093/brain/awab059 33889940 PMC8370400

[B31] Goldman S. A. Kuypers N. J. (2015). How to Make an Oligodendrocyte. Development 142 (23), 3983–3995. 10.1242/dev.126409 26628089 PMC4712837

[B32] Guo Y. Xiao P. Lei S. Deng F. Xiao G. G. Liu Y. (2008). How Is mRNA Expression Predictive for Protein Expression? A Correlation Study on Human Circulating Monocytes. Acta Biochim. Biophys. Sinica 40 (5), 426–436. 10.1111/j.1745-7270.2008.00418.x 18465028

[B33] Gutowski N. J. Newcombe J. Cuzner M. L. (1999). Tenascin-R and C in Multiple Sclerosis Lesions: Relevance to Extracellular Matrix Remodelling. Neuropathol. Appl. Neurobiol. 25 (3), 207–214. 10.1046/j.1365-2990.1999.00176.x 10417662

[B34] Haage V. Elmadany N. Roll L. Faissner A. Gutmann D. H. Semtner M. (2019). Tenascin C Regulates Multiple Microglial Functions Involving TLR4 Signaling and HDAC1. Brain Behav. Immun. 81, 470–483. 10.1016/j.bbi.2019.06.047 31271872

[B35] Hagemeier K. Brück W. Kuhlmann T. (2012). Multiple Sclerosis - Remyelination Failure as a Cause of Disease Progression. Histol. Histopathol 27 (3), 277–287. 10.14670/HH-27.277 22237705

[B36] Harauz G. Boggs J. M. (2013). Myelin Management by the 18.5-kDa and 21.5-kDa Classic Myelin Basic Protein Isoforms. J. Neurochem. 125 (3), 334–361. 10.1111/jnc.12195 23398367 PMC3700880

[B37] Hertz L. Chen Y. (2016). Editorial: All 3 Types of Glial Cells Are Important for Memory Formation. Front. Integr. Neurosci. 10, 31. 10.3389/fnint.2016.00031 27729851 PMC5037195

[B38] Hillis J. M. Davies J. Mundim M. V. Al-Dalahmah O. Szele F. G. (2016). Cuprizone Demyelination Induces a Unique Inflammatory Response in the Subventricular Zone. J. Neuroinflammation 13 (1), 190. 10.1186/s12974-016-0651-2 27550173 PMC4994223

[B39] Hughes E. G. Appel B. (2016). The Cell Biology of CNS Myelination. Curr. Opin. Neurobiol. 39, 93–100. 10.1016/j.conb.2016.04.013 27152449 PMC4987163

[B40] Jakovcevski I. Miljkovic D. Schachner M. Andjus P. R. (2013). Tenascins and Inflammation in Disorders of the Nervous System. Amino Acids 44 (4), 1115–1127. 10.1007/s00726-012-1446-0 23269478

[B41] Karus M. Denecke B. ffrench-Constant C. Wiese S. Faissner A. (2011). The Extracellular Matrix Molecule Tenascin C Modulates Expression Levels and Territories of Key Patterning Genes during Spinal Cord Astrocyte Specification. Development 138 (24), 5321–5331. 10.1242/dev.067413 22071102

[B42] Karus M. Ulc A. Ehrlich M. Czopka T. Hennen E. Fischer J. (2016). Regulation of Oligodendrocyte Precursor Maintenance by Chondroitin Sulphate Glycosaminoglycans. Glia 64 (2), 270–286. 10.1002/glia.22928 26454153

[B43] Keough M. B. Rogers J. A. Zhang P. Jensen S. K. Stephenson E. L. Chen T. (2016). An Inhibitor of Chondroitin Sulfate Proteoglycan Synthesis Promotes central Nervous System Remyelination. Nat. Commun. 7, 11312. 10.1038/ncomms11312 27115988 PMC4853428

[B44] Kiernan B. W. Götz B. Faissner A. ffrench-Constant C. (1996). Tenascin-C Inhibits Oligodendrocyte Precursor Cell Migration by Both Adhesion-dependent and Adhesion-independent Mechanisms. Mol. Cell Neurosci. 7 (4), 322–335. 10.1006/mcne.1996.0024 8793866

[B45] Kipp M. Clarner T. Dang J. Copray S. Beyer C. (2009). The Cuprizone Animal Model: New Insights into an Old story. Acta Neuropathol. 118 (6), 723–736. 10.1007/s00401-009-0591-3 19763593

[B46] Koch T. Brugger T. Bach A. Gennarini G. Trotter J. (1997). Expression of the Immunoglobulin Superfamily Cell Adhesion Molecule F3 by Oligodendrocyte-Lineage Cells. Glia 19 (3), 199–212. 10.1002/(sici)1098-1136(199703)19:3<199::aid-glia3>3.0.co;2-v 9063727

[B47] Lau L. W. Keough M. B. Haylock-Jacobs S. Cua R. Döring A. Sloka S. (2012). Chondroitin Sulfate Proteoglycans in Demyelinated Lesions Impair Remyelination. Ann. Neurol. 72 (3), 419–432. 10.1002/ana.23599 23034914

[B48] Lindner M. Heine S. Haastert K. Garde N. Fokuhl J. Linsmeier F. (2008). Sequential Myelin Protein Expression during Remyelination Reveals Fast and Efficient Repair after central Nervous System Demyelination. Neuropathol. Appl. Neurobiol. 34 (1), 105–114. 10.1111/j.1365-2990.2007.00879.x 17961136

[B49] Maeda N. Fukazawa N. Ishii M. (2010). Chondroitin Sulfate Proteoglycans in Neural Development and Plasticity. Front. Biosci. 15, 626–644. 10.2741/3637 20036837

[B50] Matsushima G. K. Morell P. (2001). The Neurotoxicant, Cuprizone, as a Model to Study Demyelination and Remyelination in the central Nervous System. Brain Pathol. 11 (1), 107–116. 10.1111/j.1750-3639.2001.tb00385.x 11145196 PMC8098267

[B51] Miller R. H. (2002). Regulation of Oligodendrocyte Development in the Vertebrate CNS. Prog. Neurobiol. 67 (6), 451–467. 10.1016/s0301-0082(02)00058-8 12385864

[B52] Miron V. E. Boyd A. Zhao J.-W. Yuen T. J. Ruckh J. M. Shadrach J. L. (2013). M2 Microglia and Macrophages Drive Oligodendrocyte Differentiation during CNS Remyelination. Nat. Neurosci. 16 (9), 1211–1218. 10.1038/nn.3469 23872599 PMC3977045

[B53] Miron V. E. Kuhlmann T. Antel J. P. (2011). Cells of the Oligodendroglial Lineage, Myelination, and Remyelination. Biochim. Biophys. Acta (Bba) - Mol. Basis Dis. 1812 (2), 184–193. 10.1016/j.bbadis.2010.09.010 20887785

[B54] Momčilović M. Stamenković V. Jovanović M. Andjus P. R. Jakovčevski I. Schachner M. (2017). Tenascin-C Deficiency Protects Mice from Experimental Autoimmune Encephalomyelitis. J. Neuroimmunology 302, 1–6. 10.1016/j.jneuroim.2016.12.001 27974153

[B55] Moritz S. Lehmann S. Faissner A. von Holst A. (2008). An Induction Gene Trap Screen in Neural Stem Cells Reveals an Instructive Function of the Niche and Identifies the Splicing Regulator Sam68 as a Tenascin-C-Regulated Target Gene. Stem Cells 26 (9), 2321–2331. 10.1634/stemcells.2007-1095 18617690

[B56] Nash B. Thomson C. E. Linington C. Arthur A. T. McClure J. D. McBride M. W. (2011). Functional Duality of Astrocytes in Myelination. J. Neurosci. 31 (37), 13028–13038. 10.1523/JNEUROSCI.1449-11.2011 21917786 PMC6623277

[B57] Nave K.-A. (2010). Myelination and Support of Axonal Integrity by Glia. Nature 468 (7321), 244–252. 10.1038/nature09614 21068833

[B58] Okuda H. Tatsumi K. Morita S. Shibukawa Y. Korekane H. Horii-Hayashi N. (2014). Chondroitin Sulfate Proteoglycan Tenascin-R Regulates Glutamate Uptake by Adult Brain Astrocytes. J. Biol. Chem. 289 (5), 2620–2631. 10.1074/jbc.M113.504787 24337573 PMC3908396

[B59] Patani R. Balaratnam M. Vora A. Reynolds R. (2007). Remyelination Can Be Extensive in Multiple Sclerosis Despite a Long Disease Course. Neuropathol. Appl. Neurobiol. 33 (3), 277–287. 10.1111/j.1365-2990.2007.00805.x 17442065

[B60] Perides G. Erickson H. Rahemtulla F. Bignami A. (1993). Colocalization of Tenascin with Versican, a Hyaluronate-Binding Chondroitin Sulfate Proteoglycan. Anat. Embryol. 188 (5), 467–479. 10.1007/BF00190141 7508696

[B61] Pesheva P. Gloor S. Schachner M. Probstmeier R. (1997). Tenascin-R Is an Intrinsic Autocrine Factor for Oligodendrocyte Differentiation and Promotes Cell Adhesion by a SulfatideMediated Mechanism. J. Neurosci. 17 (12), 4642–4651. 10.1523/JNEUROSCI.17-12-04642.1997 9169525 PMC6573339

[B62] Pu A. Stephenson E. L. Yong V. W. (2018). The Extracellular Matrix: Focus on Oligodendrocyte Biology and Targeting CSPGs for Remyelination Therapies. Glia 66 (9), 1809–1825. 10.1002/glia.23333 29603376

[B63] Ransohoff R. M. (2012). Animal Models of Multiple Sclerosis: the Good, the Bad and the Bottom Line. Nat. Neurosci. 15 (8), 1074–1077. 10.1038/nn.3168 22837037 PMC7097342

[B64] Rathjen F. G. Hodge R. (2020). Early Days of Tenascin-R Research: Two Approaches Discovered and Shed Light on Tenascin-R. Front. Immunol. 11, 612482. 10.3389/fimmu.2020.612482 33488619 PMC7820773

[B65] Reyes-Haro D. Mora-Loyola E. Soria-Ortiz B. García-Colunga J. (2013). Regional Density of Glial Cells in the Rat Corpus Callosum. Biol. Res. 46 (1), 27–32. 10.4067/S0716-97602013000100004 23760411

[B66] Roll L. Faissner A. (2019). Tenascins in CNS Lesions. Semin. Cell Developmental Biol. 89, 118–124. 10.1016/j.semcdb.2018.09.012 30287388

[B67] Sachs H. H. Bercury K. K. Popescu D. C. Narayanan S. P. Macklin W. B. (2014). A New Model of Cuprizone-Mediated Demyelination/remyelination. ASN Neuro 6 (5), 175909141455195. 10.1177/1759091414551955 PMC4187018 25290063

[B68] Shen K. Yuen T. J. (2020). *Ex Vivo* Myelination and Remyelination in Cerebellar Slice Cultures as a Quantitative Model for Developmental and Disease-Relevant Manipulations. JoVE 160. 10.3791/61044 32597864

[B69] Skripuletz T. Lindner M. Kotsiari A. Garde N. Fokuhl J. Linsmeier F. (2008). Cortical Demyelination Is Prominent in the Murine Cuprizone Model and Is Strain-dependent. Am. J. Pathol. 172 (4), 1053–1061. 10.2353/ajpath.2008.070850 18349131 PMC2276412

[B70] Sospedra M. (2018). B Cells in Multiple Sclerosis. Curr. Opin. Neurol. 31 (3), 256–262. 10.1097/WCO.000000000000563 29629941

[B71] Stark A. M. Pfannenschmidt S. Tscheslog H. Maass N. Rösel F. Mehdorn H. M. (2006). Reduced mRNA and Protein Expression of BCL-2 versus Decreased mRNA and Increased Protein Expression of BAX in Breast Cancer Brain Metastases: a Real-Time PCR and Immunohistochemical Evaluation. Neurol. Res. 28 (8), 787–793. 10.1179/016164106X110364 17288732

[B72] Steelman A. J. Thompson J. P. Li J. (2012). Demyelination and Remyelination in Anatomically Distinct Regions of the Corpus Callosum Following Cuprizone Intoxication. Neurosci. Res. 72 (1), 32–42. 10.1016/j.neures.2011.10.002 22015947 PMC3230728

[B73] Torkildsen Ø. Brunborg L. A. Myhr K.-M. Bø L. (2008). The Cuprizone Model for Demyelination. Acta Neurol. Scand. 117, 72–76. 10.1111/j.1600-0404.2008.01036.x 18439226

[B74] Tucker R. Drabikowski K. Hess J. Ferralli J. Chiquet-Ehrismann R. Adams J. (2006). Phylogenetic Analysis of the Tenascin Gene Family: Evidence of Origin Early in the Chordate Lineage. BMC Evol. Biol. 6, 60. 10.1186/1471-2148-6-60 16893461 PMC1578592

[B75] Tucker R. P. Chiquet-Ehrismann R. (2015). Tenascin-C: Its Functions as an Integrin Ligand. Int. J. Biochem. Cell Biol. 65, 165–168. 10.1016/j.biocel.2015.06.003 26055518

[B76] Ulc A. Zeug A. Bauch J. van Leeuwen S. Kuhlmann T. Ffrench-Constant C. (2019). The Guanine Nucleotide Exchange Factor Vav3 Modulates Oligodendrocyte Precursor Differentiation and Supports Remyelination in white Matter Lesions. Glia 67 (2), 376–392. 10.1002/glia.23548 30450647

[B77] Vogel C. Marcotte E. M. (2012). Insights into the Regulation of Protein Abundance from Proteomic and Transcriptomic Analyses. Nat. Rev. Genet. 13 (4), 227–232. 10.1038/nrg3185 22411467 PMC3654667

[B78] Wenk M. B. Midwood K. S. Schwarzbauer J. E. (2000). Tenascin-C Suppresses Rho Activation. J. Cell Biol 150 (4), 913–920. 10.1083/jcb.150.4.913 10953015 PMC2175281

[B79] Wheeler N. A. Fuss B. (2016). Extracellular Cues Influencing Oligodendrocyte Differentiation and (Re)myelination. Exp. Neurol. 283 (Pt B), 512–530. 10.1016/j.expneurol.2016.03.019 27016069 PMC5010977

[B80] Xiang Z. Nesterov E. E. Skoch J. Lin T. Hyman B. T. Swager T. M. (2005). Detection of Myelination Using a Novel Histological Probe. J. Histochem. Cytochem. 53 (12), 1511–1516. 10.1369/jhc.5A6704.2005 16046669 PMC3957546

[B81] Xiao Z.-c. Taylor J. Montag D. Rougon G. Schachner M. (1996). Distinct Effects of Recombinant Tenascin-R Domains in Neuronal Cell Functions and Identification of the Domain Interacting with the Neuronal Recognition Molecule F3/11. Eur. J. Neurosci. 8 (4), 766–782. 10.1111/j.1460-9568.1996.tb01262.x 9081628

[B82] Zacharias U. Leuschner R. Norenberg U. Rathjen F. G. (2002). Tenascin-R Induces Actin-Rich Microprocesses and Branches along Neurite Shafts. Mol. Cell Neurosci. 21 (4), 626–633. 10.1006/mcne.2002.1203 12504595

[B83] Zacharias U. Rauch U. (2006). Competition and Cooperation between Tenascin-R, Lecticans and Contactin 1 Regulate Neurite Growth and Morphology. J. Cell Sci 119 (Pt 16), 3456–3466. 10.1242/jcs.03094 16899820

[B84] Zhang C. Walker A. K. Zand R. Moscarello M. A. Yan J. M. Andrews P. C. (2012). Myelin Basic Protein Undergoes a Broader Range of Modifications in Mammals Than in Lower Vertebrates. J. Proteome Res. 11 (10), 4791–4802. 10.1021/pr201196e 22420465 PMC3612544

[B85] Zhang H. Jarjour A. A. Boyd A. Williams A. (2011). Central Nervous System Remyelination in Culture - A Tool for Multiple Sclerosis Research. Exp. Neurol. 230 (1), 138–148. 10.1016/j.expneurol.2011.04.009 21515259 PMC3117145

[B86] Zhang S.-C. (2001). Defining Glial Cells during CNS Development. Nat. Rev. Neurosci. 2 (11), 840–843. 10.1038/35097593 11715061

[B87] Zhao C. Fancy S. P. J. Franklin R. J. M. ffrench-Constant C. (2009). Up-regulation of Oligodendrocyte Precursor Cell αV Integrin and its Extracellular Ligands during central Nervous System Remyelination. J. Neurosci. Res. 87 (15), 3447–3455. 10.1002/jnr.22231 19739252

[B88] Zuchero J. B. Fu M.-m. Sloan S. A. Ibrahim A. Olson A. Zaremba A. (2015). CNS Myelin Wrapping Is Driven by Actin Disassembly. Developmental Cell 34 (2), 152–167. 10.1016/j.devcel.2015.06.011 26166300 PMC4519368

