# Supplementary figures and images for: Tenascins Interfere With Remyelination in an Ex Vivo Cerebellar Explant Model of Demyelination
Source: Front Cell Dev Biol. 2022 Mar 15;10:819967. doi: 10.3389/fcell.2022.819967 (PMC8965512; doi:10.3389/fcell.2022.819967)

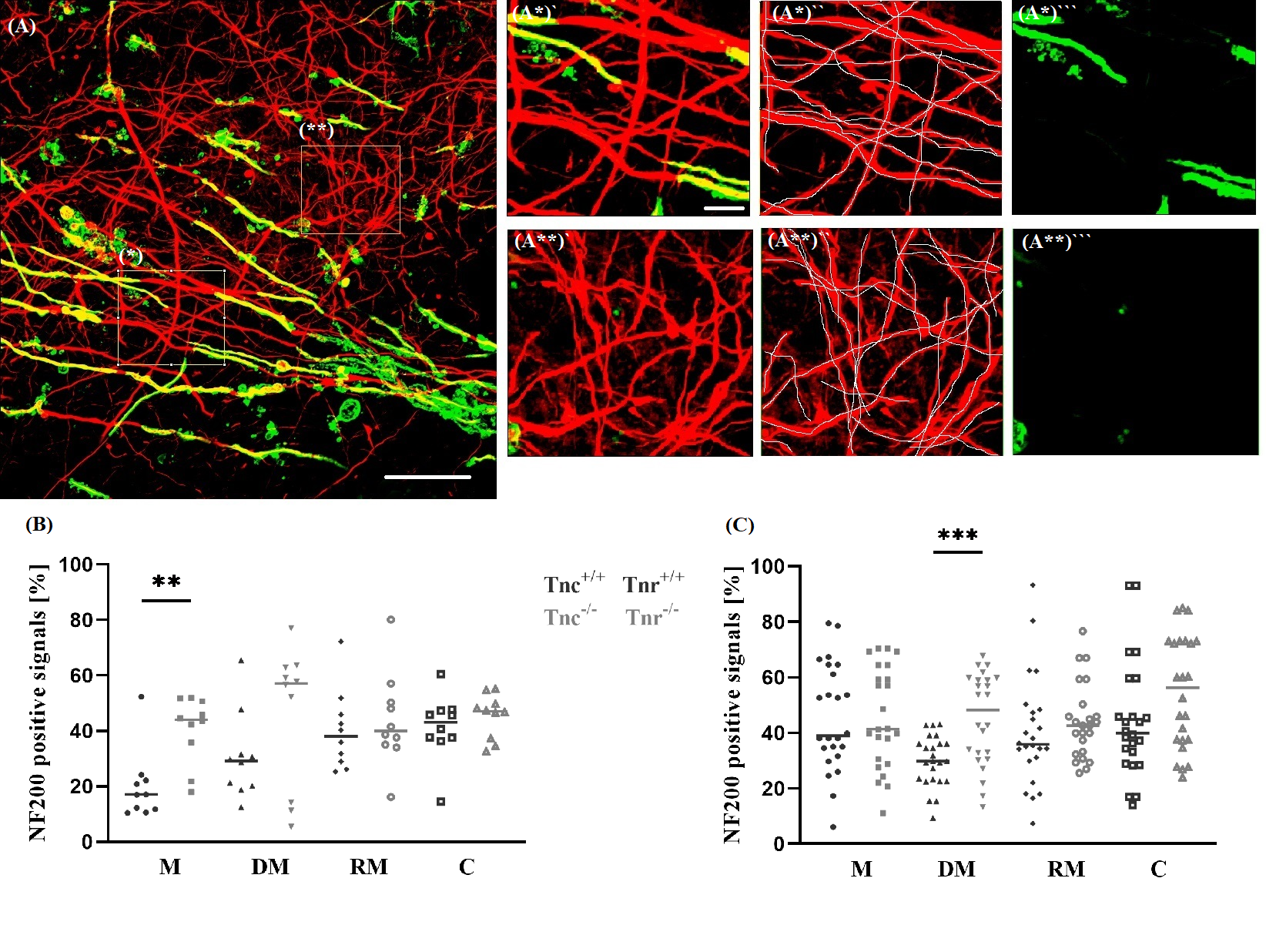

Supplement: Supplementary file 1 [file Image2.TIF]

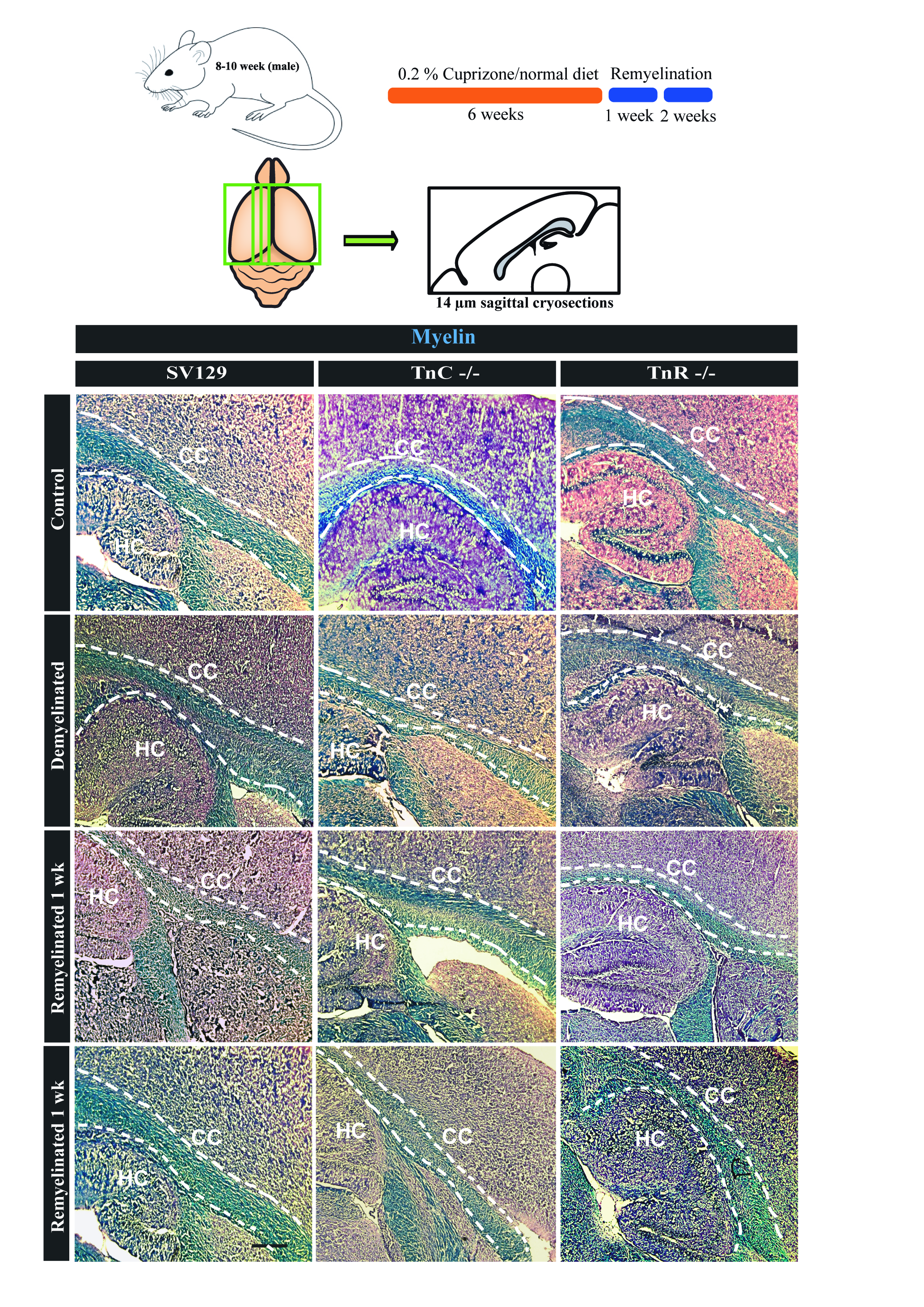

Supplement: Supplementary file 2 [file Image1.TIF]
