# Supplementary material for: Tenascins Interfere With Remyelination in an Ex Vivo Cerebellar Explant Model of Demyelination
Source: Front Cell Dev Biol. 2022 Mar 15;10:819967. doi: 10.3389/fcell.2022.819967 (PMC8965512; doi:10.3389/fcell.2022.819967)
Supplement: Supplementary file 3 [file DataSheet1.docx]

**Supplement**

**Supplemental Figure 1. (A-B) Comparison of myelin staining in the corpus callosum of SV129-wildtype, *Tnc^-/-^*- and *Tnr^-/-^*-mice under control, demyelinated and remyelinated condition. (A)** 8 until 10-week-old male mice received either a normal diet for control situation or a 0.2 % cuprizone diet to induce demyelination, after 6 weeks some mice were perfused, and some other mice received a normal diet for one or two weeks to allow for remyelination. Sagittal cryosections were used for immunohistochemistry. The corpus callosum, which is the region of interest for the following analyses, is shown in grey. **(B)** Illustration represents the LFB-PAS staining of *in-vivo* brain sections of SV129-, *Tnc^-/-^*- and *Tnr^-/-^*-mice under four conditions (control, demyelinated, one-week remyelination and two weeks remyelination). LFB stains myelin in blue and PAS axons in red. In the control group of SV129-, *Tnc^-/-^*- and *Tnr^-/-^*-brain sections a strong myelin staining (blue) appeared around the corpus callosum (CC). In *Tnr^-/-^*-sections areas of the CC with reduced myelin staining were detectable. In contrast, upon demyelination a reduced intensity of myelin staining was observable in the CC compared to the control. In all genotypes, especially in SV129- and *Tnr^-/-^*-brain sections a stronger PAS-staining was optically visible. After one week of remyelination there was no visible difference regarding the myelination grade. In contrast, after two weeks of remyelination in all genotypes a stronger intensity of the myelin staining was detectable. The LFB-PAS-staining did not distinguish the different genotypes. The Illustration of the corpus callosum was performed around the hippocampus (HC) in sagittal cryosections. Scale bar: 400 µm. 3 different animals per group and genotype were analysed.

**Supplemental Figure 2. (A, A*´-A**```, B, C) Exemplary comparison of Axon thickness in cerebellar explant cultures of *Tnc^+/+^, Tnc^-/-^,* *Tnr^+/+^* and *Tnr^-/-^* mice.** (A) *Tnc^+/+^, Tnc^-/-^,* *Tnr^+/+^* and *Tnr^-/-^* cerebellar explant cultures of the conditions myelinated (M), demyelinated (DM), remyelinated (RM) and control (C) were labelled with antibodies against NF200 (red) and MBP (green) (A, A*´, A**´) to visualize the wrapping of myelin membranes around nerve fibres. (A*, A**) Two areas were pointed out for counting the individual axons in the explants (A*, A**). Axons were first traced and then counted, but this method proved to be unsuitable for excluding axon fasciculation (A*``, A**``). NF200-positive fluorescence was measured in *Tnc^+/+^* and *Tnc^-/-^* mice (B) and in *Tnr^+/+^* and *Tnc^-/-^* mice (C) for analysing the influence of both genotypes on the availability of axonal surfaces. In myelination condition the number of axon signals was higher in *Tnc^-/-^* mice. In the case of demyelination the fraction of axon signals was increased in *Tnr^-/-^* mice. A comparable difference in *Tnc^-/-^* mice appeared not significant due to considerable scatter of the data. In all other conditions no differences were detectable. All data are provided as Mean ± SEM. Statistical significance was assessed using the two-way-ANOVA (p ≤0.05 *, p≤ 0.01 **, p≤ 0.001 ***) and Tukey´s multiple comparisons test. Scale bars: 20 µm. At least three independent experiments (N = 3) were performed, and three explants (n = 2) were analysed per experiment for each condition.
